# The mechanism of the PI3K-AKT-mTOR signaling pathway in renal cell carcinoma: current developments and future prospects

**DOI:** 10.3389/fonc.2026.1745024

**Published:** 2026-03-27

**Authors:** Can Zou, Yuxia Wang, Qiuyan Chen, Jianyou Shi, Xianbo Wu, Quan Luo

**Affiliations:** 1 School of Sports Medicine and Health, Chengdu Sport University, Chengdu, Sichuan, China; 2 Department of Pharmacy, Sichuan Academy of Medical Sciences&Sichuan Provincial People’s Hospital, Personalized Drug Therapy Key Laboratory of Sichuan Province, School of Medicine, University of Electronic Science and Technology of China, Chengdu, Sichuan, China; 3 Department of Pharmacy, Sichuan Academy of Medical Sciences & Sichuan Provincial People’s Hospital, Personalized Drug Therapy Key Laboratory of Sichuan Province, School of Medicine, University of Electronic Science and Technology of China, Chengdu, Sichuan, China; 4 West China School of Medicine, Sichuan University, The Second People’s Hospital of Chengdu Affiliated to Sichuan University, Chengdu Second People’s Hospital, Chengdu, Sichuan, China

**Keywords:** mechanism, mutations, PI3K-Akt-mTOR signaling pathway, renal cell carcinoma, therapeutics

## Abstract

Renal cell carcinoma (RCC) is a type of solid tumor with one of the highest incidences among urinary system malignancies, and its incidence continues to increase worldwide. The PI3K-AKT-mTOR signaling pathway, as a central signaling hub that regulates biological processes such as cell survival, proliferation, metabolism, and metastasis, often exhibits abnormal and sustained activation during the pathological progression of RCC. Dysregulation of this pathway synergistically promotes tumor progression through multiple mechanisms, including enhancing the survival and clonal expansion of tumor cells, inducing angiogenesis, driving metabolic reprogramming of the tumor microenvironment, and mediating treatment resistance. These pathological changes are closely associated with poor patient prognosis. Given the central role of the PI3K-AKT-mTOR signaling pathway in the pathogenesis of RCC, it has become a key target for targeted therapeutic intervention. Although multiple small-molecule inhibitors targeting this pathway have demonstrated potential for inhibiting tumor growth in preclinical studies and early-phase clinical trials, their clinical application still faces numerous challenges. Against this backdrop, combination therapy strategies offer a new approach to overcome the limitations of single-agent therapy, not only enhancing treatment efficacy but also potentially reducing the risk of drug resistance. Notably, natural products and their derivatives, due to their low toxicity, ability to modulate multiple targets, and specific inhibitory effects on cancer stem cells, are regarded as promising sensitizers in combination therapy. This article systematically reviews the pathological features of RCC and current clinical treatment strategies, with a focus on exploring the molecular regulatory mechanisms of the PI3K-AKT-mTOR signaling pathway in tumor progression, while highlighting the latest research advances in small-molecule inhibitors targeting this pathway. Integrating preclinical mechanistic studies and relevant clinical trial data, the limitations of existing agents are analyzed, and potential optimization directions are proposed, aiming to provide theoretical support and practical references for the clinical translation of precision therapy for RCC and to promote the transformation of pathway-based combination therapy models into clinical applications.

## 1 Introduction

Renal cell carcinoma (RCC) is a malignant tumor originating from the renal tubular epithelial cells, accounting for 90-95% of all kidney cancers (1). Over the past two decades, RCC incidence has been increasing at an average rate of 2% annually, with more than 200,000 new cases reported worldwide each year, predominantly in Europe and North America (2). RCC can be classified into three main subtypes based on pathological features: clear cell renal cell carcinoma (ccRCC, 75%), papillary renal cell carcinoma (pRCC, 10%), and chromophobe renal cell carcinoma (chRCC, 5%) (3, 4). Among these, ccRCC is the most common and aggressive subtype, associated with the poorest prognosis (5). It is worth noting that approximately 30% of patients are diagnosed with metastatic disease at the time of initial diagnosis (6, 7). The five-year overall survival (OS) rate is about 93% for early-stage RCC but below 15% for metastatic cases (8, 9). Furthermore, RCC often shows resistance to conventional radiotherapy and chemotherapy.

The PI3K-AKT-mTOR signaling pathway functions as a central regulatory pathway governing cell survival, proliferation, metabolism, and angiogenesis (10–12). In ccRCC, this pathway is frequently hyperactivated, driving both tumorigenesis and malignant progression while also contributing to therapeutic resistance. Despite its recognition as a highly promising therapeutic target in RCC, only a few selective inhibitors against this pathway have gained regulatory approval for clinical use (13). Moreover, with the rising incidence of RCC, the limitations of conventional therapies in improving long-term patient survival have become increasingly evident. As a key signaling hub that integrates growth factor signaling, cellular metabolism, and microenvironmental responses, aberrant activation of the PI3K-AKT-mTOR pathway plays a pivotal role in RCC initiation and progression. However, existing reviews predominantly focus on isolated mechanisms or fragmented discussions of clinical translation, lacking a systematic integration of this pathway across multiple dimensions, including genetic drivers, metabolic reprogramming, angiogenesis, apoptosis regulation, and drug resistance mechanisms. Concurrently, systematic reviews addressing the limited efficacy, feedback reactivation, and combination intervention strategies of pathway inhibitors in clinical practice remain scarce, and a comprehensive conceptual framework for understanding these complexities has yet to be established.

Accordingly, from a systems biology perspective, this review integrates the genetic basis of RCC with the aberrant activation mechanisms of the PI3K-AKT-mTOR pathway, systematically dissects its network functions in metabolic reprogramming, angiogenesis, and the regulation of apoptosis and autophagy, and establishes its central hub role in RCC initiation and progression. It comprehensively summarizes recent advances in small-molecule inhibitors and natural products targeting this pathway, comparing the therapeutic advantages and limitations of distinct intervention strategies. Furthermore, it dissects the mechanisms of intrinsic and acquired resistance involving this pathway and, building on this analysis, proposes novel combination-therapy-centered strategies to reverse resistance. Through this integrated analysis, the review seeks to establish a new theoretical framework and offer strategic guidance for precision therapy in RCC.

## 2 Characteristics of renal cell carcinoma

RCC predominantly affects individuals aged between 60 and 70, with a male-to-female prevalence ratio of approximately 1.5 to 2 (14). Key risk factors include smoking, obesity, hypertension, and metabolic syndrome (15). ccRCC, the most common subtype, exhibits distinct metabolic reprogramming involving glucose, lipid, and amino acid metabolism (16). Furthermore, ccRCC is closely linked to the tumor microenvironment (TME), which is characterized by extensive vascularization and infiltration of immune cells, reflecting the interplay between tumor biology and its surrounding environment (17, 18). Approximately 3-5% of RCC cases are associated with hereditary genetic disorders, including von Hippel-Lindau (VHL) syndrome, Phosphatase and tensin homolog (PTEN) missense tumor syndrome, Birt-Hogg-Dube syndrome, and tuberous sclerosis, among others. These syndromes often involve chromosomal abnormalities, such as the loss of chromosome 3p, a hallmark of ccRCC. This region contains key tumor suppressor genes, including VHL, PBRM1, SETD2, and BAP1, which are frequently mutated in sporadic and familial ccRCC cases. Notably, VHL mutations disrupt the regulation of hypoxia-inducible factor (HIF), leading to pathological angiogenesis and tumor growth (19, 20).

ccRCC is increasingly recognized as a metabolic disease, with dysregulated lipid and glucose metabolism central to its phenotype (21). The interplay between altered metabolism and the PI3K-AKT-mTOR pathway further accentuates tumor aggressiveness. Among these, loss or mutation of the VHL gene on chromosome 3p25–26 is the most common and pivotal oncogenic driver in ccRCC (22). The VHL gene encodes the VHL protein, a substrate recognition subunit of the ubiquitin E3 ligase complex responsible for mediating the ubiquitination and degradation of HIF, thereby suppressing tumor growth. In ccRCC, loss of VHL function results in pathological accumulation of HIF in cells (5). Elevated HIF levels increase angiogenesis and stimulate the expression of growth factors like vascular endothelial growth factor (VEGF), platelet-derived growth factor (PDGF), and epidermal growth factor (EGF). These factors bind to receptor tyrosine kinases (RTKs), aberrantly activating the PI3K-AKT-mTOR and RAS-MEK-ERK signaling pathway, driving tumor cell growth, proliferation, and metastasis (5, 23). Furthermore, the over-activated PI3K-AKT-mTOR signaling pathway exacerbates RCC onset and progression by enhancing HIF expression, creating a positive feedback loop that sustains pathway activation (24, 25).

## 3 Current status of treatment of RCC

Nephrectomy, active surveillance and radioablation are usually used to treat limited RCC (2, 14). However, recurrence or metastasis occurs in approximately 30% of patients after surgery, and over one-third of patients present with distant metastasis at the time of initial diagnosis (6, 26). Advances in genomic research, particularly through The Cancer Genome Atlas (TCGA), have transformed the treatment of advanced or metastatic RCC (mRCC), shifting from traditional cytokine therapies to targeted therapies and novel immunotherapies. The clear cell carcinoma subtype has been the focus of most clinical trials, as its histological features influence therapeutic decisions. Several targeted therapies have been approved by the US Food and Drug Administration (FDA) for advanced or mRCC, including VEGF monoclonal antibodies (e.g., Bevacizumab), tyrosine kinase inhibitors (TKIs) (e.g., Sorafenib, Sunitinib, Pazopanib, Axitinib, Lenvatinib, and Cabozantinib), and mTOR inhibitors (e.g., Temsirolimus and Everolimus) (27, 28). TKIs are anti-angiogenic agents that inhibit tumor angiogenesis by targeting vascular endothelial growth factor receptors (VEGFR), platelet-derived growth factor receptors, fibroblast growth factor receptors, and other kinases. Temsirolimus and Everolimus are rapamycin derivatives that bind to FK506-binding protein-12 (FKBP12) with high affinity, inhibiting the mTORC1 complex and thereby blocking cell growth and division. Immunotherapy, which aims to boost the immune system’s ability to target tumor cells, has made significant strides with immune checkpoint inhibitors (ICIs). ICIs such as the anti-PD-1 monoclonal antibodies Nivolumab and Pembrolizumab, the anti-PD-L1 monoclonal antibody Atezolizumab, and the anti-CTLA-4 monoclonal antibody Ipilimumab work by counteracting tumor-induced immune suppression, enhancing T-cell activation, and promoting tumor cell elimination (29, 30). Additionally, other innovative immunotherapies, including modified cytokines, cellular therapies, and cancer vaccines, are also being explored.

A risk model developed and validated by the International Metastatic RCC Database Consortium and Memorial Sloan Kettering Cancer Center classifies patients with locally advanced or mRCC into high-risk (favorable), intermediate-risk (intermediate), and low-risk (poor) groups (31, 32). According to current European Association of Urology (EAU) guidelines, first-line treatment for metastatic ccRCC typically involves a combination of TKIs and ICIs, such as Pembrolizumab + Axitinib (PEM+AXI), Cabozantinib + Nivolumab (CAB+NIV), Lenvatinib + Pembrolizumab (LEN+PEM), Ipilimumab + Nivolumab (IPI+NIV), Sunitinib, Cabozantinib, and Pazopanib (**Table 1**) (14, 33). These therapies have shown promising clinical efficacy in patients with advanced or metastatic ccRCC, significantly prolonging OS. However, the complete remission rate remains below 10%. Furthermore, with prolonged treatment, most patients develop drug resistance, experience side effects, and eventually face disease progression (30, 34). Additionally, RCC patients are burdened by the high cost of targeted therapies and novel immunotherapeutic agents. As current treatments demonstrate limited efficacy, inevitable resistance, and widespread side effects, there is a pressing need to identify new, more effective, cost-efficient drugs for RCC treatment.

**Table 1:** First-line treatment agents recommended by the EAU guidelines for metastatic ccRCC.

Risk level	Treatment standards	Alternative treatment for patients unable to receive or tolerate ICI
Favorable risk	Pembrolizumab+Axitinib(PEM+AXI) Nivolumab+Cabozantinib(NIV+CAB) Pembrolizumab+Lenvatinib(PEM+LEN)	Sunitinib Pazopanib
Intermediate and poor risk	Pembrolizumab+Axitinib(PEM+AXI) Nivolumab+Cabozantinib(NIV+CAB) Pembrolizumab+Lenvatinib(PEM+LEN) Ipilimumab+Nivolumab(IPI+NIV)	Sunitinib Cabozantinib Pazopanib

## 4 Overview of the PI3K-AKT-mTOR signaling pathway

### 4.1 The structure and function of PI3K

**Figure 1:**
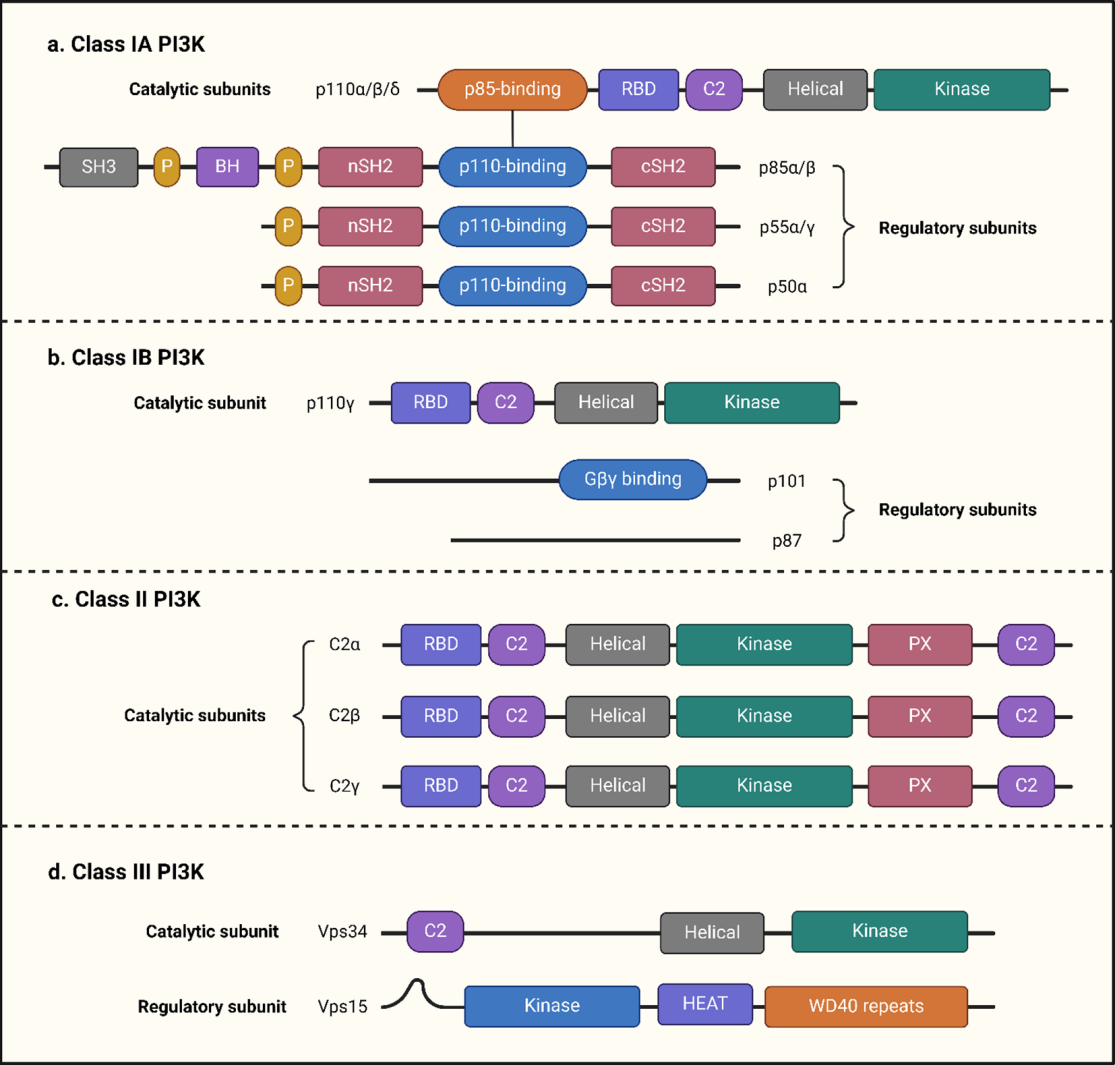
Diagrammatic representation of the PI3K domain structures. PI3K is classified into three classes (I, II, and III), with distinct catalytic and regulatory subunit architectures. Subfigure **(a)** Class IA PI3K comprises catalytic subunits (p110α/β/δ) and regulatory subunits (p85α/β, p55α/γ, and p50α). Subfigure **(b)** Class IB PI3K consists of a catalytic subunit (p110γ) and regulatory subunits (p101 and p87). Subfigure **(c)** Class II PI3K is composed of three catalytic subunits (C2α, C2β, and C2γ). Subfigure **(d)** Class III PI3K contains a catalytic subunit (Vps34) and a regulatory subunit (Vps15).

PI3K plays a crucial role as a downstream effector of G protein-coupled receptors (GPCRs) and RTKs, actively participating in intracellular signaling pathways critical for cell survival and growth (**Figure 1**) (35, 36). Class I PI3Ks directly generate phospholipids for signaling, whereas class II and III PI3Ks are more involved in intracellular membrane transport processes. Class I PI3Ks are heterodimers composed of a catalytic subunit (p110) and a regulatory subunit (p85). Class I is further divided into two subclasses: class I A (PI3Kα, β, and δ) and class I B (PI3Kγ). Class I A PI3Ks are activated by GPCRs, RAS, and RTKs, while class I B PI3K is activated exclusively by GPCRs. Both classes convert phosphatidylinositol-4,5-bisphosphate (PIP2) to phosphatidylinositol-3,4,5-trisphosphate (PIP3) upon activation, with PIP3 acting as a second messenger to recruit cytoplasmic proteins (37). The catalytic subunit of Class I PI3Ks (p110) has three isoforms: p110α, p110β, and p110δ, which are encoded by the genes PIK3CA, PIK3CB, and PIK3CD, respectively. Among these, PIK3CA mutations are the most common in various cancers and are linked to poorer prognosis (38). The regulatory subunit (p85) contains SH2 and SH3 domains that preferentially bind phosphorylated tyrosine residues. It has five isoforms: p85α, p85β, p55α, p55γ, and p50α. Of these, p85α, p55α, and p50α are encoded by the PIK3R1 gene; the PIK3R2 gene encodes p85β, and the PIK3R3 gene encodes p55γ. Class I B PI3K comprises the catalytic subunits p110γ and regulatory subunits p101 and p87. Class I PI3K is implicated in cancer and functions by phosphorylating PIP2 to generate PIP3. In contrast, class II and III PI3Ks have distinct structures and functions. Class II PI3Ks consist of three catalytic subunits, C2α, C2β, and C2γ, without a regulatory subunit, and primarily phosphorylate phosphatidylinositol to produce phosphatidylinositol triphosphate (39). Class III PI3Ks consist of a catalytic subunit (Vps34) and a regulatory subunit (Vps15), mainly involved in protein and vesicle trafficking, phosphorylating phosphatidylinositol to phosphatidylinositol triphosphate (40, 41).

### 4.2 The structure and function of AKT

**Figure 2:**
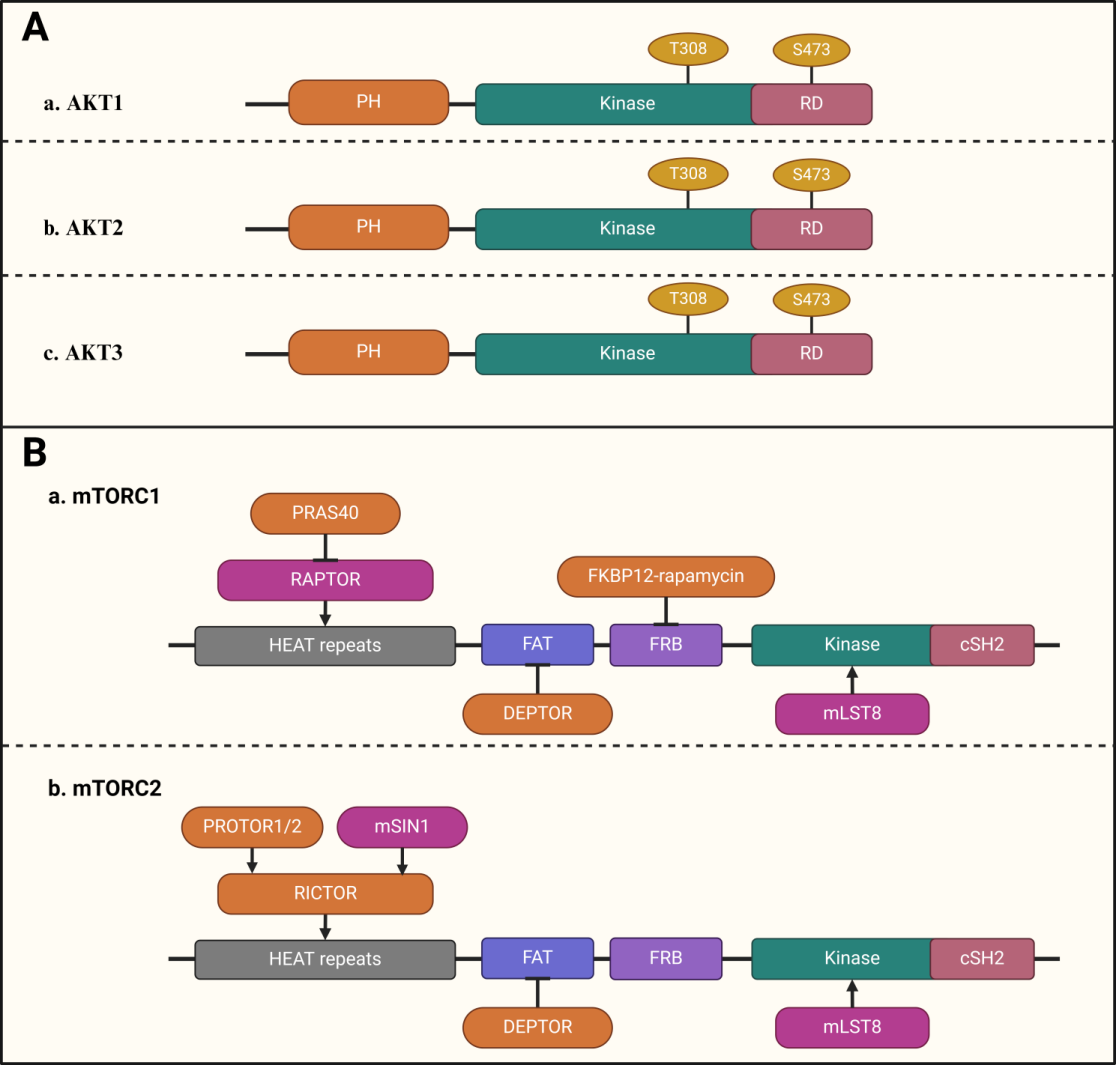
Diagrammatic representation of the AKT and mTOR domain structures. **(A)** (a–c). Schematic diagram of AKT. AKT can be divided into three isoforms: AKT1 (a), AKT2 (b), and AKT3 (c). All AKT isoforms consist of three distinct domains: the N‑terminal PH domain, the central catalytic domain, and the C‑terminal regulatory domain. **(B)** (a–b). Schematic diagram of mTOR. mTOR exists in two structurally and functionally distinct complexes: mTORC1 (a) and mTORC2 (b).

Protein kinase B (PKB), commonly referred to as AKT, is a highly conserved serine/threonine kinase within the AGC kinase family and is a key protein in the PI3K signaling pathway, playing a critical role in cell survival and apoptosis. In mammals, AKT can be divided into three isoforms, AKT1, AKT2, and AKT3, encoded by the PKBα, PKBβ, and PKBγ genes (42). AKT1 is the most widely expressed isoform, crucial for cell growth and survival in various tissues. AKT2 is mainly found in insulin-sensitive tissues like muscle, liver, and adipocytes, where it participates in mitochondrial-mediated apoptosis, metabolism, migration, and invasion. AKT3 is predominantly expressed in the brain and testis (41, 43, 44). All AKT isoforms share 85% amino acid sequence homology and are composed of three distinct structural domains: the PH domain at the N-terminal, which binds PIP3 and enables AKT’s interaction with the cell membrane; the central catalytic domain, which is kinase-active and phosphorylates a broad range of substrates; and the C-terminal regulatory domain, which is modulated by phosphorylation from other kinases (**Figure 2**) (45–47). Full activation of AKT requires dual phosphorylation. The major phosphorylation sites of AKT1/2/3 are located at Thr308/Thr309/Thr305 in the catalytic domain (phosphorylated by PDK1) and Ser473/Ser474/Ser472 in the regulatory domain (phosphorylated by mTORC2) (46, 48). Upon activation, AKT translocates from the membrane to other cellular compartments and phosphorylates downstream targets such as mTOR, glycogen synthase kinase-3, FOXO, caspase-3, and cyclin D1. These proteins are involved in regulating cell growth, apoptosis, the cell cycle, and angiogenesis.

### 4.3 The structure and function of mTOR

mTOR, a serine/threonine protein kinase localized in the cytoplasm, comprises six distinct structural domains: the HEAT repeats region, FAT domain, FRB domain, kinase catalytic domain, and FATC regulatory domain (**Figure 2**) (49). This evolutionarily conserved signaling hub integrates nutrient, energy, and growth factor cues to orchestrate fundamental cellular processes, including protein synthesis, autophagy, cell cycle progression, and metabolic reprogramming, thereby governing cell fate decisions critical for organismal development and homeostasis. mTOR exists in two structurally and functionally distinct complexes: mTORC1 and mTORC2.mTORC1 includes the catalytic subunit mTOR, the regulatory subunit Raptor (regulatory-associated protein of mTOR), the structural subunit mLST8 (mammalian lethal with Sec13 protein 8), the negative regulatory subunit PRAS40 (40 kDa proline-rich AKT substrate), and DEPTOR (DEP domain-containing protein) (50). The downstream substrates of mTORC1, such as 4EBPs, S6K1, and unc-51-like kinase 1 (ULK1), are markers for detecting mTORC1 activity (51). mTORC1 is sensitive to rapamycin and promotes cell growth and metabolism by inducing protein and lipid synthesis, ribosome biogenesis, and reducing autophagy (52–54). The mTORC2 consists of the catalytic subunit mTOR, the structural subunit Rictor(rapamycin-insensitive companion of mTOR), the positive regulatory subunits mLST8, mSIN1(mammalian stress-activated protein kinase interacting protein), PROTOR1/2(protein observed with Rictor 1/2) and the negative regulatory subunit DEPTOR (55). Downstream kinases of mTORC2 include AKT, protein kinase C, and serum/glucocorticoid-regulated kinase (SGK). mTORC2 is insensitive to rapamycin and mainly regulates cell survival, proliferation, and cytoskeletal remodeling (52). The human Ras homolog enriched in the brain (RHEB) is a GTPase that regulates the mTOR signaling pathway. RHEB activates mTORC1 by promoting its binding to its substrate, enhancing mTORC1 activity (56, 57). When RHEB binds to GDP, it inhibits mTORC1; when bound to GTP, mTORC1 is activated (58). Under normal conditions, the tuberous sclerosis complex 1/2 (TSC1/2) complex promotes the conversion of RHEB-GTP to inactive RHEB-GDP, thereby inhibiting mTORC1. However, phosphorylation of TSC2 by AKT inactivates the TSC1/2 complex, leading to mTORC1 activation (59).

### 4.4 PI3K-AKT-mTOR signaling pathway

**Figure 3:**
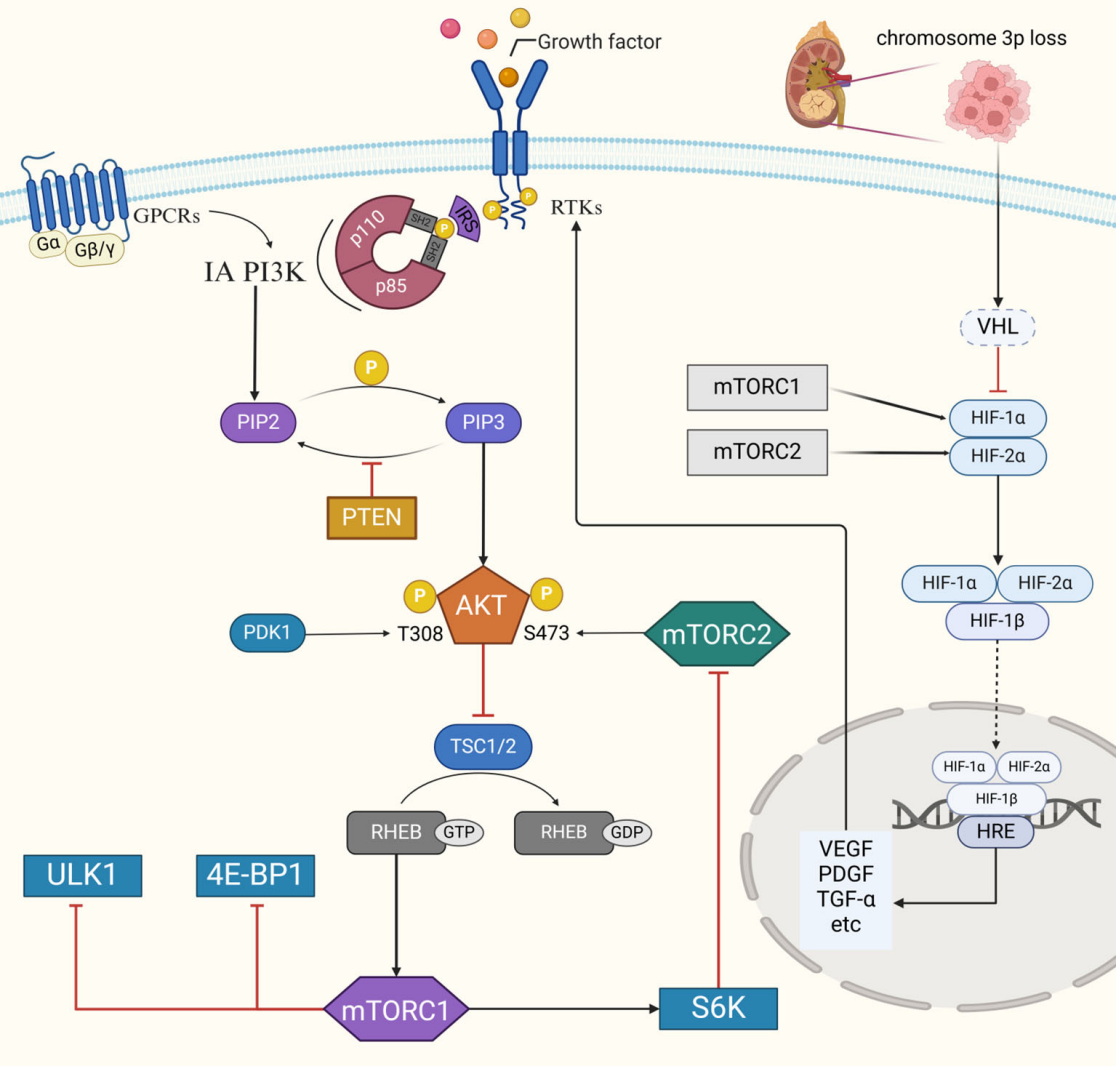
Schematic overview of the PI3K-AKT-mTOR signaling pathway. Stimulation of various RTKs and GPCRs by extracellular signals leads to PI3K being activated, thereby converting PIP2 to PIP3. AKT phosphorylated atThr308 and Ser473 by PDK1 and mTORC2 respectively. The phosphorylation and activation of AKT then leads to the mTORC1 complex, which affects many downstream targets. Black arrows indicate protein activation, red arrows indicate protein inhibition.

The PI3K-AKT-mTOR signaling pathway plays a crucial role in maintaining the biological functions of cells (both normal and tumor cells), including metabolism, cell survival, growth, proliferation, and angiogenesis (37, 45, 60). PI3K can be activated by the interaction of ligands, including VEGF, PDGF, insulin-like growth factor (IGF), and EGF, with RTKs or GPCRs (**Figure 3**) (61, 62). When the ligand binds to the corresponding membrane receptor, it induces the recruitment of class I PI3K proteins through junction proteins such as insulin receptor substrate (IRS), which phosphorylates the D3 hydroxyl group of phosphatidylinositol, thereby converting PIP2 to PIP3 (63). PTEN negatively regulates this process by dephosphorylating PIP3 back to PIP2, thus attenuating PI3K signaling (64, 65). PIP3 acts as a second messenger to recruit AKT from the cytoplasm to the cell membrane by binding to the pleckstrin homology (PH) domain of AKT. Phosphorylation at Thr308 and Ser473 by PDK1 and mTORC2, respectively, activates AKT (66–68). Activated AKT can directly activate mTORC1 by phosphorylating the Ser2448 site or indirectly by inhibiting TSC1/2 (40). mTORC1 then phosphorylates and activates downstream effectors, including eIF4E-binding protein 1 (4E-BP1) and p70 ribosomal protein S6 kinase (p70S6K), which regulate cellular anabolic growth through mRNA translation, protein synthesis, lipid synthesis, and glucose metabolism (69). mTORC2 is activated by growth factors in a PI3K-dependent manner. Once activated, mTORC2 phosphorylates and activates AGC kinases such as AKT and SGK1, which regulate cell growth, metabolism, and autophagy (52, 70).

## 5 Mechanism of PI3K-AKT-mTOR signaling pathway in renal cell carcinoma

The PI3K-AKT-mTOR signaling pathway serves as a critical regulator of cellular processes such as survival, proliferation, angiogenesis, metabolism, and resistance to therapy. Its overactivation is a common feature in solid tumors, including ccRCC. In ccRCC patients, dysregulation of this pathway, characterized by hyperactivation—is strongly linked to aggressive tumor behavior and unfavorable clinical outcomes.

### 5.1 Genetic alterations of the PI3K-AKT-mTOR signaling pathway in RCC

Various genetic alterations are implicated in the onset and progression of ccRCC, primarily involving chromosome 3p. These alterations include mutations in genes regulating cytosolic oxygen sensing (e.g., VHL) and those involved in chromatin remodeling and DNA methylation (e.g., PBRM1, SETD2, and BAP1) (22, 71, 72). VHL is the most commonly mutated gene in ccRCC, and its complete loss, through genetic and/or epigenetic mechanisms, is considered the earliest and most fundamental oncogenic event in ccRCC development (73, 74). However, individuals with VHL germline mutations develop ccRCC after a longer latency period (>30 years), and VHL-null mice do not spontaneously develop ccRCC (75–77). These observations suggest that VHL mutations alone are insufficient to induce ccRCC, and additional genetic or epigenetic events are necessary. VHL mutations do not directly influence clinical outcomes. PBRM1, SETD2, and BAP1 encode tumor suppressor proteins involved in chromatin and histone regulation, indicating that epigenetic dysregulation is crucial in ccRCC progression. Mutations in PBRM1, SETD2, and BAP1 (38%, 13%, and 11%, respectively, in TCGA-RCC) are associated with aggressive clinical features in ccRCC (78, 79). Furthermore, large-scale studies, including the TCGA, have shown frequent mutations in the PI3K-AKT-mTOR signaling pathway in ccRCC. Approximately 28% of ccRCC cases exhibit mutations in the PI3K-AKT-mTOR signaling pathway, with common alterations including PIK3CA mutations, PTEN dysfunction, TSC mutations, and mTOR mutations, all contributing to pathway dysregulation (80–82).

#### 5.1.1 Genetic alterations in the PI3K gene

The PIK3CA gene, which encodes the PI3K catalytic subunit p110α, is one of the most frequently mutated oncogenes in various cancers, with mutations and gene amplifications observed across multiple tumor types (83). Activating mutations in PIK3CA were first identified in 2004 and are commonly found in cancers such as breast, ovarian, endometrial, and cervical cancer (84). PIK3CA mutations are mainly gain-of-function missense mutations with classical mutation sites located in the helical structural domain (E542/E545/Q546) and the kinase structural domain (H1047) (85, 86). Clinically, elevated PIK3CA expression correlates with tumor invasion and poor patient prognosis. The mutation frequency of PIK3CA in cancer has been reported to range from 11-14% (87). In ccRCC, PIK3CA mutations or amplifications occur in 2-5% of cases and are mutually exclusive with mutations in other pathway components (72, 82). Activating mutations in PIK3CA lead to increased PIP3 levels, promoting the recruitment of PIP3-binding proteins, such as AKT isoforms, to the plasma membrane.

#### 5.1.2 Genetic alterations in the PTEN gene

As a key negative regulator of the PI3K-AKT-mTOR signaling pathway, PTEN influences the cell growth, proliferation, apoptosis, and metabolism of ccRCC. At the same time, it is also one of the most frequently mutated tumor suppressor genes in tumors. Dysfunction of PTEN, due to inactivating mutations, homozygous or heterozygous deletions, or epigenetic alterations, results in elevated PIP3 levels, which subsequently lead to the overactivation of the PI3K-AKT-mTOR signaling pathway. Cancer-associated mutations are distributed throughout PTEN, with the most common mutation (R130Q/G) located in the lipid phosphatase catalytic region (88–90). PTEN mutations are typically loss-of-function mutations, occurring in 1-5% of ccRCC cases (72, 82). Loss of PTEN function enhances the expression of immunosuppressive cytokines, reducing tumor T-cell infiltration and promoting resistance to therapy. While PTEN mutations are relatively rare in ccRCC, research has shown that PTEN expression is frequently reduced in most ccRCC cases (91). PIK3CA and PTEN mutations rank as the second and third most common oncogenic mutations in cancer, after p53 mutations (92). PTEN tumor suppressor activity is highly dose-dependent, meaning even subtle changes in PTEN function can significantly impact cancer susceptibility and tumorigenesis.

#### 5.1.3 Genetic alterations in the TSC1/2 gene

The TSC1/2 complex acts as a crucial negative regulator of the mTORC1 signaling pathway, and its encoded proteins interact to form a heterodimer that inhibits the activation of mTORC1, thereby integrating upstream signals from energy sensors, nutrient availability (amino acids), and growth factor signaling (PI3K/AKT) to coordinating anabolic processes, including protein synthesis, ribosome biogenesis and cell cycle processes. The proteins encoded by these genes interact to form heterodimers that inhibit mTORC1 activation. In cells with inactivated TSC1 or TSC2 alleles, mTORC1 becomes overactivated, increasing cell growth, tumorigenesis, and extensive metabolic reprogramming. Mutations in TSC1/2 are associated with tuberous sclerosis, a condition that predisposes individuals to ccRCC. TSC1/2 mutations occur in approximately 2-5% of ccRCC cases (72, 93). TSC1/2 mutations lead to increased levels of RHEB-GTP, which activates the mTORC1 signaling pathway (94). Furthermore, inactivation of TSC1/2 mutations increases HIF translation, promoting tumor progression. Analysis of the Cancer Genome Database has also identified a recurrent point mutation in RHEB (Y35N) in ccRCC, which leads to mTORC1 hyperactivation by conferring resistance to GAP activity in TSC2 (95, 96). Thus, RHEB mutations represent an additional mechanism for the activation of mTORC1.

#### 5.1.4 Genetic alterations in the mTOR gene

Activating mutations in mTOR have been found in many various types of cancers, and they are higher in RCC and endometrial cancer (95). Ghosh et al. demonstrated that mutations in the FAT structural domain of the mTOR gene are present in RCC, and Rheb mutations in RCC patients also lead to increased mTORC1 activity (97). In ccRCC, the mutations in mTOR are usually missense mutations (98, 99). Approximately 5-6% of ccRCC patients harbor mTOR mutations, which tend to cluster in key regulatory regions such as the FAT and kinase domains, leading to mTORC1 overactivation (72, 82). While these overactive mTOR mutants remain sensitive to rapamycin, mutations in the FKBP-FRB domain of mTOR can impair the binding of Temsirolimus and Everolimus, potentially inducing resistance (100). mTORC1 is activated in cancer cells through various genetic events, including mutations in PIK3CA, inactivating mutations or deletions in PTEN, activating mutations or amplifications in any of the three AKT isoforms, and inactivating mutations or deletions in TSC1/2 (101). Notably, ccRCC patients who benefit most from treatment with mTORC1 inhibitors, such as Everolimus and Temsirolimus, often carry mutations in mTOR, PI3K, TSC1/2 (102, 103).

#### 5.1.5 Genetic alterations in the AKT gene

AKT plays a central role in many signaling pathway, and its overexpression and phosphorylation-driven activation are common events in human cancers. However, AKT gene mutations are relatively rare in these cancers (104, 105). Acquired missense mutations and amplifications have been identified in genes encoding the three isoforms of AKT, a protein involved in regulating cell survival, proliferation, apoptosis, and glycogen metabolism. One hotspot mutation, AKT1 E17K, significantly increases the affinity of AKT for PtdIns(4,5)P2, which activates AKT1 and promotes downstream signaling through its pathological localization to the plasma membrane (106, 107). Other activating mutations include AKT1 E49K and AKT3 G171R. While mutations or amplifications of AKT are rare in ccRCC, AKT activation is commonly driven by PTEN deletion.

Alterations in these genes are largely mutually exclusive, and most mutations are in oncogenes, resulting in loss of function. ccRCC also exhibits extensive intra-tumor heterogeneity, which may lead to underestimation of mutation frequencies in current assays (108, 109). The intra-tumor heterogeneity of ccRCC also complicates the identification and validation of biomarkers. No single predictive biomarker has been validated for clinical guidance in ccRCC. Genetic variants in PI3K-AKT-mTOR signaling pathway genes may contribute to resistance to RCC treatments.

### 5.2 PI3K-AKT-mTOR signaling pathway and angiogenesis in RCC

Angiogenesis refers to the process of forming new blood vessels from pre-existing ones, enabling the delivery of oxygen and nutrients to the body’s tissues. This process is physiological and halts after reaching adulthood. However, persistent abnormal angiogenesis is a hallmark of cancer, promoting tumor growth and metastasis (110). In addition, angiogenesis is directly or indirectly involved in tumor escape from the immune system. In RCC, angiogenesis is a key driver of tumorigenesis and disease progression. ccRCC is highly vascularized, exhibiting complex neovascularization and overexpression of VEGF and other pro-angiogenic factors (2, 111). VEGF drives sustained angiogenesis in cancer, forming abnormal, hyperpermeable, and disorganized blood vessels. Upregulation of VEGF expression and signaling suggests that angiogenesis plays a central role in ccRCC. VEGF-A, a potent angiogenic factor, binds to receptors on endothelial cells, particularly VEGFR2, to stimulate tumor cell growth, proliferation, migration, and angiogenesis (112, 113). The mainstay of RCC treatment involves anti-angiogenesis therapies targeting VEGF/VEGFR. FDA-approved targeted ccRCC drugs are classified into two categories: angiogenesis inhibitors, which target VEGF or VEGFR, and mTOR inhibitors, which block both tumor cell growth and angiogenesis. However, hypoxia resulting from tumor vascular degeneration during anti-angiogenic therapy may increase the expression of various pro-angiogenic factors.

Activation of the PI3K-AKT-mTOR signaling pathway plays a key role in angiogenesis. In ccRCC, this pathway increases VEGF expression and stimulates angiogenesis through both HIF-α-dependent and -independent mechanisms. Additionally, hypoxic conditions and VHL dysfunction also enhance VEGF expression, and VEGF binding to the corresponding receptors can directly activate the PI3K-AKT-mTOR signaling pathway, thereby inducing angiogenesis. The PI3K-AKT-mTOR signaling pathway is also involved in angiogenesis by regulating the expression of other angiogenic factors, such as nitric oxide and angiopoietin (114). Angiogenesis is critical for RCC progression, aiding the recruitment of endothelial cells. mTOR acts as a key regulator of cellular anabolism and catabolism in these cells. Inhibition of the mTOR pathway can block VEGF-mediated angiogenesis and cell growth by decreasing VEGF production and secretion, as well as reducing VEGFR2 signaling. Furthermore, EGFR and PDGF contribute to the hypoxic response, promoting endothelial cell vascularization (115). In addition, abnormal angiogenesis controls immune cell infiltration and promotes hypoxia, thereby enhancing the immunosuppressive effects of pro-angiogenic factors on immune cells (116).

### 5.3 PI3K-AKT-mTOR signaling pathway and metabolic reprogramming in RCC

As a metabolic disease, RCC has various metabolic disorders (117). In ccRCC, these abnormalities include reprogramming of glucose, lipid, and amino acid metabolism, with glycolysis being the most prominent alteration (16, 118). Metabolic reprogramming is a key tumorigenic feature that supports the rapid proliferation of cancer cells by meeting their increased demands for essential cellular building blocks, such as proteins, DNA, and membranes. In ccRCC, metabolic reprogramming enables tumor cells to survive in nutrient- and oxygen-deprived environments, escape immune surveillance, and maintain their ability to proliferate and metastasize (119). Metabolic reprogramming in ccRCC is primarily driven by the inactivation of the VHL gene and the activation of the PI3K-AKT-mTOR signaling pathway. Key genes involved in regulating this reprogramming include VHL, PTEN, AKT, mTOR, TSC1/2, and Myc (120–122). TCGA studies have revealed significant metabolic reprogramming in ccRCC, with upregulation of glycolysis, the pentose phosphate pathway, fatty acid synthesis, and glutamine metabolism, and downregulation of the tricarboxylic acid (TCA) cycle (16, 72).

#### 5.3.1 Glucose metabolism

In normal cells, glucose catabolism produces pyruvate, broken down and metabolized in the TCA cycle, releasing large amounts of ATP during oxidative phosphorylation. Under hypoxia, normal cells switch to anaerobic glycolysis, converting pyruvate to lactate. However, in cancer cells, glucose is preferentially fermented to lactate for energy production, even in the presence of oxygen and functional mitochondria. This metabolic alteration is known as the Warburg effect, or aerobic glycolysis (123). Aerobic glycolysis is crucial in the early stages of carcinogenesis, as it increases lactate production and reduces TCA cycle activity. ccRCC follows the classic Warburg effect (124). Glucose uptake is primarily mediated by glucose transporter-1 (GLUT-1), and high GLUT-1 expression in ccRCC cells, compared to normal tissue, indicates increased glucose consumption (125). Furthermore, metabolomics, transcriptomics, and proteomics studies have shown that ccRCC cells overexpress enzymes involved in glycolysis, such as hexokinase (HK), phosphoglycerate kinase, pyruvate kinase, and lactate dehydrogenase while expressing lower levels of pyruvate dehydrogenase (PDH) and pyruvate carboxylase, both of which promote glycolysis and inhibit the entry of pyruvate into the TCA cycle (126–128). The PI3K-AKT-mTOR signaling pathway plays a key role in insulin signaling and glucose homeostasis. In cancer cells, it induces the expression of key genes involved in aerobic glycolysis, including GLUT1, HK, and LDHA. Abnormalities in the PI3K/AKT pathway promote a metabolic shift towards aerobic glycolysis, leading to increased glucose uptake and enhanced glycolysis. AKT signaling can stimulate metabolic changes by directly phosphorylating key metabolic enzymes or indirectly regulating transcription factors to promote aerobic glycolysis (129). Activated AKT increases glucose uptake via GLUT-1 and enhances the Warburg effect (130). Additionally, AKT can inhibit the TCA cycle in the Warburg effect by PDK1, which inhibits PDH (45). Studies have shown that the downregulation of TCIRG1, a glycolysis-associated biomarker, inhibits aerobic glycolysis in ccRCC through the AKT/mTOR signaling pathway (131).

In the TCA cycle of ccRCC, citrate and cis-aconitate levels are significantly increased, while malate, fumarate, and succinate levels are significantly reduced (117, 126). Additionally, glucose is metabolized through the pentose phosphate pathway (PPP). This pathway converts glucose-6-phosphate from glycolysis into fructose-6-phosphate, producing nicotinamide adenine dinucleotide phosphate (NADPH) and ribose-5-phosphate, which are critical for lipid and nucleotide biosynthesis (117, 132). In ccRCC, the upregulation of the PPP boosts NADPH production, helping to alleviate oxidative stress, maintain redox balance, and protect cells from damage caused by reactive oxygen species (ROS) (133). Glucose-6-phosphate dehydrogenase (G6PD), the rate-limiting enzyme in PPP, is significantly elevated in ccRCC cells and is associated with poor prognosis. There is reciprocal crosstalk between the PI3K/AKT signaling pathway and PPP metabolism (134). The PI3K-AKT-mTOR signaling pathway directs glucose carbon flux into both the oxidative and non-oxidative branches of the PPP, facilitating ribose production for nucleotide synthesis. Furthermore, activation of the PI3K/AKT pathway stabilizes G6PD, promoting PPP activity, enhancing metabolic activity and driving cancer cell progression. The PI3K-AKT-mTOR signaling pathway also stimulates the expression of HIF-1α, which regulates cancer cell metabolism by increasing glucose uptake through both glycolysis and PPP. The PI3K-AKT-mTOR-HIF-1α signaling pathway is typically regarded as a unified mechanism that coordinates glycolysis regulation (135). Moreover, excessive lactate accumulation increases Rheb-GTP levels by inhibiting TSC2 binding to Rheb, activating the mTORC1 signaling pathway and promoting tumor progression. The activation of mTOR is closely linked to metabolic reprogramming in RCC (136).

#### 5.3.2 Lipid metabolism

The high level of lipid accumulation in ccRCC indicates dysregulated lipid metabolism, which is closely linked to increased invasiveness and metastatic potential. In ccRCC, lipid metabolism is characterized by enhanced fatty acid and lipid synthesis and reduced utilization and oxidation, resulting in elevated levels of fatty acids, cholesterol, and triglycerides. High fatty acid levels are observed in ccRCC cells alongside the downregulation of β-oxidation pathways (124). Lipid droplets, a hallmark of ccRCC, store fatty acids, cholesteryl esters (CEs), and phospholipids near the endoplasmic reticulum. This storage is crucial for maintaining endoplasmic reticulum integrity and lipid homeostasis by preventing fatty acid toxicity, which in turn supports ccRCC cell survival (137). The PI3K/AKT signaling pathway reprograms cellular metabolism in cancer cells by enhancing the activity of nutrient transporters and metabolic enzymes to meet the anabolic demands of rapidly proliferating cells. Silencing ALDH3A2, a key regulator of lipid metabolism, activates the PI3K/AKT signaling pathway, promoting lipid accumulation and tumor progression in ccRCC (138). Dysregulation of lipid metabolism is also closely linked to activation of the mTOR pathway. Overexpression of ECHS1, a key enzyme in fatty acid metabolism, inhibits RCC cell growth and migration by suppressing mTOR activation, whereas ECHS1 is downregulated in ccRCC and negatively correlates with the ccRCC phenotype and mTOR pathway activation (139). These findings suggest that targeting the PI3K-AKT-mTOR signaling pathway can reduce lipid metabolism and accumulation, thereby inhibiting ccRCC progression. Furthermore, the PI3K-AKT-mTOR signaling pathway influences lipid metabolism by activating sterol regulatory element-binding proteins, which promote adipogenesis and increase triacylglycerol synthesis (140). The abnormal accumulation of CEs in ccRCC is driven by VHL mutations, leading to HIF-α stabilization and upregulation of the PI3K-AKT-mTOR-SREBP signaling pathway through various growth factors and transmembrane receptors (141). Both HIF-1α and HIF-2α are positively correlated with the PI3K-AKT-mTOR signaling pathway, with HIF-1α mainly regulating glycolysis and HIF-2α governing lipid metabolism, ribosome biogenesis, and angiogenesis (142, 143).

Several key enzymes involved in fatty acid metabolism play significant roles in ccRCC, including fatty acid synthase (FASN), carnitine palmitoyltransferase 1A (CPT1A), ATP citrate lyase (ACLY), acetyl-CoA carboxylase (ACC), and stearoyl-CoA desaturase 1 (SCD1) (144). Overexpression of FASN in ccRCC promotes fatty acid synthesis and is positively correlated with tumor aggressiveness and poor prognosis (145, 146). CPT1A, a key enzyme in fatty acid β-oxidation, is downregulated in ccRCC, impairing fatty acid oxidation and causing lipid accumulation in the cytoplasm (147). ACLY, which converts citrate from the TCA cycle into acetyl-CoA, provides a substrate for fatty acid synthesis. The highly active PI3K/AKT signaling pathway directly regulates fatty acid synthesis by phosphorylating and activating ACLY, thereby initiating *de novo* lipid synthesis (148, 149). ACC, a rate-limiting enzyme in fatty acid synthesis, converts acetyl-CoA into malonyl-CoA. Inhibition of ACC with TOFA (5-tetradecyloxy-2-furoic acid) induces cell cycle arrest and apoptosis by suppressing the PI3K-AKT-mTOR signaling pathway, thereby inhibiting ccRCC cell growth (150). AMP-activated protein kinase (AMPK), a critical sensor of cellular energy balance, inhibits fatty acid and cholesterol resynthesis by phosphorylating and inhibiting ACC, thus maintaining cellular energy homeostasis and protecting cells from metabolic stress. Dysregulation of the ACC/AMPK signaling pathway promotes fatty acid synthesis in ccRCC (151). TSC1/2, involved in the AMPK-mTOR nutrient and energy sensing pathway, inhibits mTORC1 activity in response to energy deprivation by activating TSC1/2 protein heterodimers. SCD1, highly expressed in ccRCC, increases the desaturation of endogenous fatty acids. Inhibitors of SCD1 block RCC cell growth and induce apoptosis, and combining SCD1 inhibitors with mTOR inhibitors shows synergistic anti-tumor activity (152). Zhang et al. demonstrated a positive feedback loop between HIF-2α and SCD1, with SCD1 overexpression in ccRCC required for PI3K/AKT pathway activation. Additionally, activation of the PI3K/AKT pathway mediates SCD1-regulated HIF-2α overexpression, further promoting ccRCC (153).

#### 5.3.3 Amino acids metabolism

In ccRCC, glutamine, tryptophan, and arginine metabolism is reprogrammed to support cancer progression. Glutamine is particularly essential for ccRCC, with activation of the PI3K/AKT signaling pathway driving its utilization in metabolic pathways, thereby enhancing cell growth. Glutamine catabolism generates reducing agents such as reduced glutathione (GSH), NADPH, and α-ketoglutarate, which help mitigate oxidative stress. Proteomic and metabolomic studies have shown that elevated glutamine activates the GSH/GSSG pathway, counteracting oxidative stress and ROS (154, 155). In ccRCC, the balance between GSH and GSSG is tightly regulated. Additionally, glutamine contributes to fatty acid synthesis via reductive carboxylation and promotes glutamate production, a key mechanism for neutralizing ROS (119). Glutamate is converted into α-ketoglutarate (α-KG) by glutamate dehydrogenase 1 (GLUD1). α-KG enters the TCA cycle, supporting lipid synthesis in ccRCC (156). The PI3K-AKT-mTOR signaling pathway regulates both glutamine metabolism and the TCA cycle (157, 158). Inhibition of the mTOR pathway reduces protein translation, including glucose and amino acid transporters, which limits extracellular nutrient uptake. Wang et al. showed that GLUD1 levels are decreased in ccRCC and that GLUD1 suppresses ccRCC development by inhibiting PI3K-AKT-mTOR activation (159). Furthermore, low GLUD1 expression correlates with poor prognosis and reduced sensitivity to TKIs in ccRCC.

Arginine succinate synthase 1 (ASS1) is the key enzyme responsible for the endogenous production of arginine, synthesized from citrulline through the urea cycle. However, ASS1 is either not expressed or expressed at low levels in ccRCC, resulting in a high dependence on exogenous arginine for tumor cell survival and proliferation (160, 161). Tryptophan is metabolized mainly via the kynurenine pathway, and indoleamine 2,3-dioxygenase 1 (IDO1) is the main rate-limiting enzyme of this pathway. In ccRCC, overexpression of IDO1 leads to reduced tryptophan levels and activation of the kynurenine pathway. Upregulation of tryptophan metabolism increases immunosuppression, promoting immune evasion by ccRCC cells (126, 162). IDO1 regulates the PI3K-AKT-mTOR signaling pathway in cancer cells. For example, IDO1 and kynurenine metabolites promote PI3K/AKT activation in colon cancer epithelium, inhibiting apoptosis and supporting cell growth (163). In addition, Liu et al. demonstrated that IDO1 promotes cardiomyocyte hypertrophy by activating the PI3K-AKT-mTOR-S6K1 signaling pathway (164). These findings suggest that the PI3K-ATK-mTOR signaling pathway may similarly regulate tryptophan metabolism in ccRCC.

### 5.4 Effects of the PI3K-AKT-mTOR signaling pathway on cell growth, apoptosis and autophagy in RCC

A balance between cell growth and cell death is crucial for maintaining cellular homeostasis. This balance is disrupted in malignant tumors, where uncontrolled proliferation and evasion of apoptosis are hallmark features of cancer (165). Apoptosis, or programmed cell death, allows the removal of abnormal or unwanted cells in a controlled manner. The Bcl-2 protein family plays a central role in regulating apoptosis, with pro-apoptotic proteins (e.g., Bax, Bak) and anti-apoptotic proteins (e.g., Bcl-2, Bcl-xL) governing cell survival. In many cancers, increased expression of anti-apoptotic proteins inhibits the activation of pro-apoptotic proteins, thereby promoting cancer cell survival and resistance to apoptotic signaling (166, 167). The PI3K-AKT-mTOR signaling pathway is a complex signaling network activated in various malignancies, including RCC. It promotes cancer cell survival, proliferation, migration, and cell cycle progression while inhibiting apoptosis. Upon activation, AKT transduces anti-apoptotic signals by phosphorylating key proteins involved in cell survival and proliferation, such as Bax, Bcl-2, BAD, FOXO, GSK3, MDM2, and caspase-9 (168, 169). AKT also regulates cell growth by controlling the cell cycle. mTORC1 activation enhances protein, lipid, and nucleotide synthesis while reducing autophagy through downstream effectors like p70S6K1, 4EBP-1, and SREBP-1, thereby supporting cell survival, proliferation, and growth. Meanwhile, mTORC2 activation regulates protein kinases, including AKT, further promoting cell survival and proliferation (170, 171). Mutations or deletions in PTEN, a key negative regulator of the PI3K/AKT pathway, also contribute to increased cell growth and reduced apoptosis. For instance, Zhang et al. found that the leukotriene B4 receptor (LTB4R) is highly expressed in ccRCC, correlating with poorer prognosis. LTB4R promotes ccRCC cell growth, migration, and invasion while inhibiting apoptosis by regulating the AKT/mTOR pathway (172). Similarly, Yang et al. found that PLAUR contributes to the development of Sunitinib resistance in ccRCC (173). Further studies revealed that PLAUR promotes ccRCC proliferation and migration by activating the PI3K-AKT-mTOR signaling pathway while inhibiting cell cycle progression and apoptosis (174).

Autophagy, a key cellular recycling process, involves the degradation and elimination of damaged or dysfunctional organelles and proteins. It plays a dual role in RCC. Generally, autophagy suppresses tumor progression by reducing oxidative stress, maintaining genomic stability, and clearing dysfunctional proteins in RCC (175). The PI3K-AKT-mTOR signaling pathway is a key negative regulator of autophagy. In cancer cells, activation of this pathway inhibits autophagy, enabling them to survive in nutrient-deprived environments and continue proliferating. mTORC1 is a central regulator of autophagy in cancer cells, where its activation promotes growth and inhibits autophagy by interacting with ULK1 and autophagy-related protein 13, thus suppressing the ULK1 complex. Consequently, inhibiting mTOR is one of the most effective methods for inducing autophagy. Hydroxychloroquine, a known autophagy inhibitor, was shown in a phase I/II clinical trial involving RCC patients to potentiate the anti-cancer effects of the mTOR inhibitor Everolimus (176). Grimaldi and Li et al. also showed that chloroquine could enhance the anti-cancer effects of Everolimus and Sunitinib on RCC by inhibiting autophagy and inducing apoptosis in RCC cells (177, 178). Autophagy regulators also modulate the PI3K-AKT-mTOR and AMPK/mTOR signaling pathway. Activation of PI3K/AKT promotes cell survival by inhibiting apoptosis and suppressing autophagy while driving cell cycle progression through mTORC1 activation. For example, Gong et al. showed that curcumin-induced apoptosis and autophagy in RCC cells by inhibiting the AKT/mTOR pathway, an effect reversible by the autophagy inhibitor 3-MA (179). Antonaci et al. showed that dimethyl sulfide induced autophagy in Caki-1 kidney cells by inhibiting the PI3K-AKT-mTOR-p70S6K pathway (180). Furthermore, Que et al. showed capsaicin inhibited migration, invasion, and epithelial-mesenchymal transition of RCC cell lines 786-O and CAKI-1 by inducing AMPK/mTOR-mediated autophagy (181). Similarly, Zhang et al. demonstrated that thymoquinone induced autophagy via the AMPK/mTOR pathway, inhibiting RCC cell metastasis (182). Thus, the PI3K-AKT-mTOR signaling pathway and autophagy are pivotal in regulating RCC progression.

## 6 Additional mechanisms affecting the PI3K-AKT-mTOR signaling pathway in renal cell carcinoma

### 6.1 Crosstalk signal pathway

The VHL-HIF and PI3K-AKT-mTOR signaling pathway exhibit extensive crosstalk, with both axes contributing to the pathogenesis of ccRCC (**Figure 3**) (183). Overexpression of growth factors like VEGF, EGF, and IGF activates the PI3K-AKT-mTOR signaling pathway by binding to RTKs on tumor cells, regulating HIF-α expression and translation, and causing its accumulation (184–186). In ccRCC, VHL gene mutations or deletions impair HIF-α degradation, resulting in its stabilization, accumulation, and nuclear translocation. Activated HIF-α drives angiogenesis, cell growth, migration, vasculogenesis, and metabolic reprogramming (187). Moreover, HIF-α upregulates growth factors such as VEGF, PDGF, EGF, and IGF, further activating the PI3K-AKT-mTOR signaling pathway. This positive feedback loop stabilizes HIF-α, induces growth factor expression, and perpetuates RTK activation.

HIF, an oxygen-sensitive transcription factor, consists of HIF-α and HIF-β subunits (188). Among HIF-α subunits, HIF-2α is a key driver of ccRCC initiation and progression (189, 190). The FDA approved Belzutifan, the first HIF-2α inhibitor, in August 2021 for treating VHL-associated RCC and other VHL-related tumors (191). Preclinical studies suggest combining HIF-2α inhibitors with checkpoint inhibitors may suppress tumor growth by enhancing T-cell infiltration, modulating myeloid cell activity, and altering chemokine expression. HIF-α expression is regulated by both mTOR and AKT, with HIF-1α depending on mTORC1, mTORC2, and AKT3, while HIF-2α relies on mTORC2 and AKT2 (192). mTORC2 activation stabilizes HIF-2α and enhances PI3K/AKT-driven cell growth and survival. Additionally, TSC1/2 inactivation promotes HIF translation (193). mTOR and HIF-α pathway activation are observed in PTEN-deficient ccRCC, where VHL gene deletion regulates mTORC1 through the VHL-HIF-REDD1 (194). Loss of VHL function hyperactivates mTOR, driving tumor progression and worsening prognosis in ccRCC patients.

The VHL-HIF and PI3K-AKT-mTOR axes form an intricate network that drives tumorigenesis, angiogenesis, and migration in ccRCC. These pathways play pivotal roles in cell cycle regulation, with mTORC1 specifically modulating cyclin D1 expression via phosphorylation of 4E-BP1 and p70S6K. Dysregulated cyclin D1 is a hallmark of VHL function loss in ccRCC (195). Additionally, long non-coding RNAs may mediate crosstalk between these axes and are significantly associated with ccRCC prognosis.

### 6.2 microRNA

Emerging as pivotal regulators of RCC pathogenesis, microRNAs (miRNAs) orchestrate gene expression post-transcriptionally by binding to their target mRNAs (196). Acting as both oncogenes and tumor suppressors (197), miRNAs modulate key components of the PI3K-AKT-mTOR signaling pathway across various cancers, while this signaling pathway reciprocally influences miRNA biogenesis and function (198). In ccRCC, miRNAs drive tumorigenesis, invasion, angiogenesis, and therapeutic responses, underscoring their critical roles in disease progression. Moreover, miRNAs hold significant potential as biomarkers for early diagnosis, prognosis, and predictive therapeutic strategies (199, 200). The intricate interplay between miRNAs and the PI3K-AKT-mTOR signaling pathway governs multiple biological processes fundamental to ccRCC development.

Several miRNAs have been identified as regulators of the PI3K-AKT-mTOR signaling pathway in RCC, highlighting their pivotal roles in tumor progression and therapeutic responses. Huang et al. revealed that miR-205-5p expression is downregulated in ccRCC, where it inhibits cell growth, migration, invasion, and promotes apoptosis by targeting VEGFA and the PI3K-AKT-mTOR signaling pathway (201). Similarly, Xu et al. demonstrated that miR-182-5p acts as a tumor suppressor by negatively regulating AKT activity and inducing G1-phase arrest through inhibition of the AKT/FOXO3a signaling pathway (202). Cui et al. identified mTOR as a direct target of miR-99a, which induces cell cycle arrest and suppresses tumorigenicity in RCC both *in vitro* and *in vivo* (203). In contrast, miR-21, a well-characterized oncomiR, is overexpressed in RCC, promoting tumor growth, invasion, and poor prognosis by downregulating PTEN, which subsequently activates AKT and mTOR (204, 205). Fan et al. reported that miR-22, which is downregulated in ccRCC, suppresses tumor growth and invasion by directly targeting PTEN (206). Additionally, miR-144 inhibits RCC cell proliferation and induces S/G2-phase cell cycle arrest by targeting mTOR (207). Recent evidence suggests that miRNAs contribute to drug resistance mechanisms in RCC by modulating the PI3K-AKT-mTOR signaling pathway. This underscores their potential as therapeutic targets, providing opportunities to enhance treatment efficacy and overcome resistance in RCC.

## 7 Targeting the PI3K-AKT-mTOR signaling pathway to treat renal cell carcinoma

Targeting the PI3K-AKT-mTOR signaling pathway offers a promising strategy for treating RCC due to its pivotal role in the disease’s pathogenesis. Current therapeutic approaches that inhibit this pathway can be broadly categorized into two groups: pathway-specific inhibitors and treatments based on natural or synthetic compounds. Key therapeutic agents include PI3K inhibitors, AKT inhibitors, mTOR inhibitors, and dual PI3K/mTOR inhibitors. Although several small-molecule inhibitors have shown promise in preclinical studies, only a limited number have progressed to clinical application. Notably, Everolimus and Temsirolimus are FDA-approved therapies for RCC. For a comprehensive overview of inhibitors targeting the PI3K-AKT-mTOR signaling pathway, including their clinical development status and trial results, see **Table 2** in Chapter Seven.

**Table 2:** Overview of inhibitors in clinical trials.

Therapy	Target	Agents	Structure	Setting	Species	Status	Highlights	Key clinical endpoints	Dosing	Main conclusions	ClinicalTrials. gov identifiers	Reference
PI3K inhibitors	PI3K	Buparlisib(BKM120)	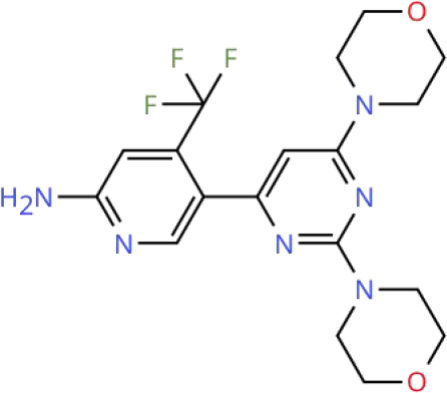	Phase IB	RCC	Completed	A study of Bevacizumab combined with Buparlisib in patients with metastatic RCC	• MTD and DLTs: MTD of Buparlisib was 80 mg/day. Two DLT cases in the 100 mg cohort included psychiatric toxicities (depression, suicidal ideation, anorexia), rash/pruritus, and elevated lipase levels. Two additional DLT cases in the 80 mg expansion cohort presented with elevated lipase levels with depression, and rash/pruritus. • Efficacy: ① ORR: 13% (4/30 patients; 95% CI: 4%-31%), all PR; ② DCR: 63% (19/30 patients), including PR and SD; ③ Median TTF: 4 months (95% CI: 2–9 months), MTD cohort: 3 months; ④ Median OS: Not reached; MTD cohort: 13 months (95% CI: 4 months to not reached). • Biomarker Analysis: ① PIK3CA mutation: 2/9 patients (22%) had the activating mutation (H1047R), with 42% tumor reduction (TTF: 13 months) and 16% tumor reduction (TTF: 9 months), respectively. ② Metabolic biomarkers: Elevated fasting blood glucose correlated with ORR (P = 0.04), serving as a potential predictive biomarker for efficacy.	Design: 3 + 3 dose escalation. Buparlisib: Oral 60, 80, 100 mg/day. Bevacizumab: intravenous 10 mg/kg once every 2 weeks. Cycle: 28 days. Duration: Continued until disease progression, intolerable toxicity, or patient withdrawal.	• Safety and Tolerability: Buparlisib (80 mg/day) plus bevacizumab (10 mg/kg biweekly) showed a manageable safety profile in patients with mRCC. The most common AEs included elevated liver enzymes, hypertension, and increased lipase. • Preliminary Efficacy: The ORR was 13% (95% CI: 4%-31%), and 50% of patients achieved SD. Patients harboring PIK3CA mutations derived more significant benefit. • Biomarkers: Elevated fasting blood glucose may serve as a predictive biomarker for therapeutic efficacy.	NCT01283048	(215)
	PI3K	SF1126	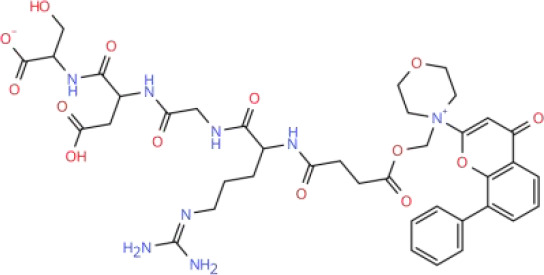	Phase I	RCC	Completed	A study of SF1126 in patients with metastatic RCC	• Safety: DLTs: None observed; Main AEs: Grade 1–2 gastrointestinal reactions (nausea, vomiting, diarrhea). • Efficacy: 1 patient with mRCC resistant to mTORC1 inhibitors achieved SD at a dose of 840 mg/m², with a duration of 84 weeks (21 cycles). • Pharmacokinetics: Exposure (Cmax, AUC0-t) was dose-proportional. The mean half-life of SF1126 was 1.0-2.4 hours.	Route: IV infusion. Dose: 90–1110 mg/m² (days 1 and 4 of a 28-day cycle). Schedule: Repeated weekly.	• SF1126 demonstrated a favorable tolerability profile in patients with RCC, with no DLTs observed. The maximum administered dose was 1110 mg/m². Exposure to the active metabolite SF1101 was dose-dependent, and effective therapeutic concentrations were achieved at doses ≥ 140 mg/m². • Long-term disease stabilization (84 weeks) was observed in patients with RCC, and clinical benefit may be achieved particularly in patients with resistance to mTORC1 inhibitors. • Pharmacodynamic data support the specific inhibition of the PI3K/mTORC pathway by SF1126, with minimal effects on normal tissues, providing a rationale for subsequent combination therapies.	NCT00907205	(219)
AKT inhibitors	AKT	Perifosine	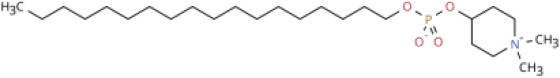	Phase II	RCC	Completed	A study of Perifosine in patients with advanced RCC following failure of VEGF-targeted therapy	• Efficacy: ① ORR: Perifosine 228: 4% (1 PR); Perifosine 231: 10% (5 PR; 13% in Arm A, 6% in Arm B); Combined analysis: 8.1%. ② SD: Perifosine 228: 46%; Perifosine 231: 32% (28% in Arm A, 39% in Arm B); Combined analysis: 36%. ③ Median PFS: Perifosine 228: 14.2 weeks (95% CI: 7.7-21.6 weeks); Perifosine 231: 14 weeks (95% CI: 12.9-20.7 weeks; 14.1 weeks in Arm A, 14 weeks in Arm B); Combined analysis: 14 weeks. ④ 12-week PFS rate: Perifosine 228: 46%; Perifosine 231: 41% in Arm A, 44% in Arm B.	Perifosine: Oral 100 mg/day.	• Efficacy: Perifosine demonstrated modest single-agent activity in patients with advanced RCC who had failed VEGF-targeted therapy, but was not superior to existing second-line treatments. • Safety: Perifosine was well tolerated, with toxicities mainly being mild-to-moderate gastrointestinal symptoms and fatigue.	NCT00498966	(225)
	AKT	MK-2206	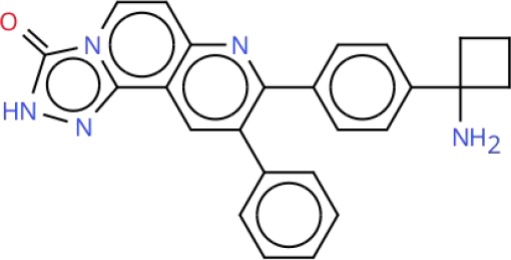	Phase II	RCC	Terminated	A study comparing MK-2206 and Everolimus in patients with advanced RCC resistant to VEGF-targeted therapy	• Efficacy: ① PFS: MK-2206 group: Median 3.68 months (95% CI: 1.77-5.75); Everolimus group: Median 5.98 months (95% CI: 5.03-not reached); Comparison: No significant difference (P = 0.27). ② ORR: MK-2206 group: 1 CR, 3 PR (ORR 13.8%); Everolimus group: No CR/PR. ③ PD: ​MK-2206 group: 44.8%; Everolimus group: 14.3%. Comparison: MK-2206 group significantly higher. ④ OS: MK-2206 group: Median 23.5 months (95% CI: 10.7-37.4); Everolimus group: Median 15.7 months (95% CI: 6.5-not reached), Comparison: No significant difference (P = 0.66). • Safety: MK-2206 arm: Common AEs: Maculopapular rash (79.3%), hyperglycemia (69%), fatigue (62.1%); Grade 3–4 AEs: Rash (27.6%), hyperglycemia (24.1%); Dose adjustment rate: 37.9%. Everolimus arm: Common AEs: Fatigue (78.6%), hyperlipidemia (64.3%), hyperglycemia (64.3%); Grade 3–4 AEs: Rare; Dose adjustment rate: 21.4%. • Genomic Analysis: PD Cohort: 57.1% of patients had deleterious mutations or deletions in TP53 or ATM genes; Non PD Cohort: No such mutations. • Pathway/Biomarkers: No activating mutations in PI3K pathway or other predictive biomarkers for therapeutic efficacy observed.	MK-2206: Oral 200 mg qw. Everolimus: Oral 10 mg qd.	• Efficacy: MK-2206 was not superior to Everolimus in PFS and was associated with a higher risk of disease progression. However, a subset of patients (13.8%) achieved deep responses (CR/PR) with MK-2206, suggesting that AKT inhibitors may be effective in a specific patient population. • Safety: MK-2206 was associated with more pronounced rash and hyperglycemia, necessitating more dose adjustments. • Genomic Markers: Mutations in DNA repair genes including TP53 and ATM were associated with early disease progression, suggesting that aberrations in the DNA repair pathway may predict a more aggressive tumor phenotype.	NCT01239342	(227)
mTOR inhibitors	mTOR	Vistuserib(AZD2014)	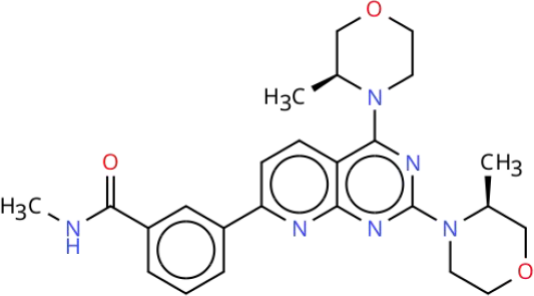	Phase II	ccRCC	Terminated	A study comparing AZD2014 and Everolimus in patients with metastatic ccRCC following failure of VEGF-targeted therapy	• Efficacy: ① PFS: AZD2014 group: Median 1.8 months; Everolimus group: Median 4.6 months, Comparison: HR = 2.8 (95% CI: 1.2-6.5, p=0.01). ② OS: AZD2014 group: Median 6.2 months; Everolimus group: Median 16.7 months, Comparison: HR = 3.1 (95% CI: 1.1-8.4, p=0.02). ③ ORR: AZD2014 group: 4%, Everolimus group: 13% (p=0.3). ④ PD: AZD2014 group: 69%; Everolimus group: 13% (p<0.001). • Safety: Grade 3–4 AEs Incidence: AZD2014 group: 35%; Everolimus group: 48% (p=0.3). Discontinuation Rate Due to AEs: AZD2014 group: 4%; Everolimus group: 4%. • Pharmacokinetics: AZD2014 Plasma Concentrations: Consistent with therapeutic range from Phase I study. Complete Analysis: Not performed (early trial discontinuation).	AZD2014: Oral 50 mg bid. Everolimus: Oral 10 mg qd.	• Although AZD2014 demonstrated an acceptable safety and pharmacokinetic profile, both PFS and OS were significantly inferior to Everolimus in patients with VEGF-refractory metastatic ccRCC.	NCT01793636	(239)
	mTOR	Sapanisertib(TAK-228)	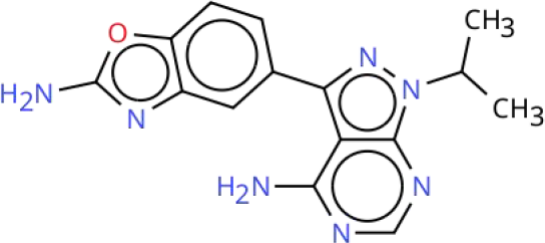	Phase II	ccRCC	Completed	A study comparing Sapanisertib (monotherapy or combination with PI3Kα inhibitor TAK-117) and Everolimus in patients with advanced ccRCC following failure of VEGF-targeted therapy	• Efficacy: ① PFS: No significant difference in median PFS among three groups (Everolimus: 3.8 months, Sapanisertib: 3.6 months, Sapanisertib + TAK-117 group: 3.1 months, HR = 1.33 and 1.37, respectively; P>0.05). ② ORR: Everolimus group: 16.7%; Sapanisertib group: 0%; Sapanisertib + TAK-117 group: 7.1%. ③OS: No significant difference in median OS among three groups (Everolimus: 22.4 months, Sapanisertib: 16.2 months, Sapanisertib + TAK-117 group: 18.1 months, P>0.05). • Safety: Discontinuation Rate Due to TEAEs: ​Sapanisertib monotherapy group: 28.1%; Sapanisertib + TAK-117 group: 29.0%; Everolimus group: 15.6%; Higher in Sapanisertib groups (mono/combo) than Everolimus group. Common TEAEs: Sapanisertib groups (mono/combo): Nausea, vomiting, fatigue; Everolimus group: Stomatitis, diarrhea.	Sapanisertib: Oral 30 mg qw. Sapanisertib + TAK-117: Oral 4 mg Sapanisertib + 200 mg TAK-117, 3 days/week. Everolimus: Oral 10 mg qd.	• Sapanisertib monotherapy or in combination with TAK-117 was not superior to Everolimus in efficacy and was associated with poorer tolerability. Dual mTORC1/2 inhibition, with or without PI3Kα inhibition, may not represent an effective therapeutic strategy for advanced ccRCC following resistance to VEGF-targeted therapy.	NCT03097328	(242)
Dual PI3K/mTOR inhibitors	PI3K/mTOR	BEZ235	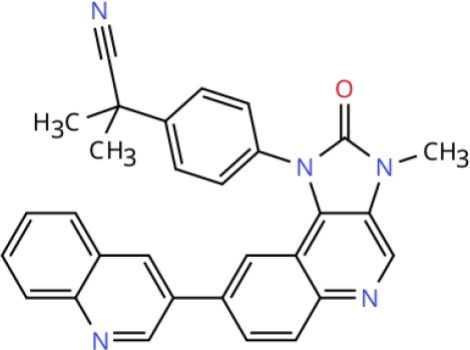	Phase IB	RCC	Terminated	A study of BEZ235 in patients with advanced RCC	• Safety: All dose groups had DLTs. Grade 3 fatigue, rash; intolerable Grade 2 nausea, vomiting, and mucositis. Discontinuation Rate Due to TEAEs: 50% of patients • Efficacy: Evaluable Patients: 5, Efficacy Distribution: SD: 2, PD: 3, Objective Responses: No CR/PR.	Design: 3 + 3 dose escalation. BEZ235: Oral 200, 300, or 400 mg bid. Duration: Until disease progression or intolerable toxicity.	• Safety: BEZ235 showed significant toxicity at all tested doses. The MTD and recommended phase II dose could not be determined, and the study was terminated early due to poor tolerability. • Efficacy: No objective responses were observed, and only 2 patients achieved SD, indicating no significant antitumor activity of the agent.	NCT01453595	(249)
	PI3K/mTOR	Apitolisib(GDC-0980)	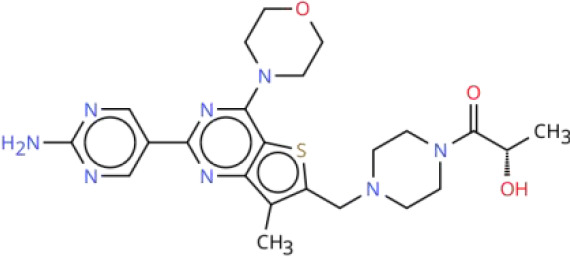	Phase II	RCC	Completed	A study comparing Apitolisib and Everolimus in patients with metastatic RCC following failure of VEGF-targeted therapy	• Efficacy: PFS: ① Apitolisib group: Median 3.7 months; Everolimus group: Median 6.1 months, Comparison: Significantly shorter in Apitolisib group (HR = 2.12, P<0.01). ② OS: Apitolisib group: Median 16.5 months; Everolimus group: Median 22.8 months, Comparison: Trend favoring Everolimus (HR = 1.77, P = 0.06). ③ ORR: Apitolisib group: 7.1%; Everolimus group: 11.6%, Comparison: No significant difference (P = 0.48). • Safety: Grade 3–4 AEs Incidence: Apitolisib group: 74%; Everolimus group: 44%; Comparison: Higher in Apitolisib group. Predominant AEs: Hyperglycemia, rash. Discontinuation Rate Due to AEs: Apitolisib group: 31%; Everolimus group: 12%; Comparison: Higher in Apitolisib group. • Biomarkers: ① VHL Gene Mutation: Everolimus group: Associated with improved efficacy; Apitolisib group: No association with efficacy. ② High HIF-1α Expression: Correlated with improved PFS in both treatment groups (Everolimus and Apitolisib).	Apitolisib: Oral 40 mg qd. Everolimus: Oral 10 mg qd.	• Efficacy: In patients with mRCC with progression on VEGF-targeted therapy, Apitolisib was significantly inferior to the mTORC1-selective inhibitor Everolimus, as evidenced by shorter PFS and greater toxicity. • Safety: The high toxicity of Apitolisib (especially hyperglycemia and rash) may limit its clinical use, leading to insufficient treatment exposure. • Biomarkers: VHL gene mutation and high HIF-1α expression were associated with the efficacy of Everolimus, whereas no such association was observed with Apitolisib.	NCT01442090	(251)

MTD, Maximum Tolerated Dose; DLTs, Dose-Limiting Toxicities; ORR, Objective Response Rate; PR, Partial Responses; DCR, Disease Control Rate; SD, Stable Disease; TTF, Time to Failure; OS, Overall Survival; AEs, Adverse Events; PFS, Progression-Free Survival; CR, Complete Response; PD, Progressive Disease; TEAEs, treatment-related adverse events.

To visualize the functional sites of the molecules, key atoms/groups are color coded as follows: blue: nitrogen (N); red: oxygen (O); green: fluorine (F); brown: sulfur (S); black: carbon (C) and hydrogen (H) backbone.

### 7.1 Development of targeted inhibitors for PI3K-AKT-mTOR in RCC

#### 7.1.1 PI3K inhibitors

PI3K inhibitors are categorized into two main types based on their pharmacokinetics and interaction with the ATP-binding cleft: pan-PI3K inhibitors and isoform-specific PI3K inhibitors. To date, the FDA has approved four PI3K inhibitors for cancer therapy: Copanlisib (pan-PI3K inhibitor), Idelalisib (PI3Kδ inhibitor), Duvelisib (dual PI3Kγ/PI3Kδ inhibitor), and Alpelisib (PI3Kα inhibitor). Copanlisib (BAY80-6946), approved in 2017, is the only pan-PI3K inhibitor for cancer treatment, specifically for relapsed or refractory follicular lymphoma following at least two prior systemic therapies (208). Idelalisib (CAL-101), approved in 2014, targets relapsed or refractory chronic lymphocytic leukemia (CLL), follicular B-cell non-Hodgkin’s lymphoma (NHL), and small lymphocytic lymphoma (SLL) (209). Similarly, Duvelisib (IPI145) received FDA approval in 2018 for relapsed or refractory CLL and SLL (210, 211). Alpelisib (BYL719), approved in 2019, is used with fulvestrant to treat HR-positive, HER2-negative, PIK3CA-mutated advanced or metastatic breast cancer and has shown promising activity in PIK3CA-mutated malignancies (212, 213). Umbralisi, a dual PI3Kδ/CK1ϵ inhibitor, was briefly approved in 2021 for relapsed or refractory marginal zone lymphoma and follicular lymphoma but was withdrawn in 2022 due to safety concerns (214). Other inhibitors, including Buparlisib (BKM120), Serabelisib (INK1117), and Tenalisib (RP6530), are under clinical investigation. Additionally, several inhibitors, such as LY294002, SF1126, and TGX211, are being evaluated in RCC clinical trials, offering potential therapeutic strategies for targeting the PI3K-AKT-mTOR signaling pathway.

##### 7.1.1.1 Buparlisib (BKM120)

Buparlisib (BKM120), an oral pan-PI3K inhibitor, has demonstrated a favorable safety profile and notable anti-tumor activity across various solid tumors (**Table 2**). Clinical evidence indicates a strong correlation between Buparlisib exposure and PI3K pathway inhibition. A phase IB clinical trial assessed the efficacy and safety of Buparlisib combined with Bevacizumab in patients with mRCC (215). Results showed that the combination regimen consisting of Buparlisib at its maximum tolerated dose of 80 mg daily plus Bevacizumab (10 mg/kg every two weeks) was well-tolerated and had a favorable safety profile. The most common treatment-related adverse events (TEAEs) were elevated hepatic transaminases (including aspartate transaminase and alanine transaminase), fatigue, and hypertension. Notably, psychiatric adverse events (AEs) (e.g., anxiety and depression) occurred in 43% of patients, necessitating close and focused monitoring. In terms of anti-tumor activity, this regimen demonstrated preliminary efficacy in mRCC patients who had progressed after prior VEGF-targeted therapy, yielding an objective response rate (ORR) of 13% (95% confidence interval [CI]: 4%-31%). Of particular interest, patients with PIK3CA gene mutations appeared to derive greater benefit from the combination. Additionally, preliminary analysis revealed a positive correlation between elevated fasting blood glucose levels and ORR, suggesting that fasting blood glucose may serve as a potential biomarker for predicting clinical response.

##### 7.1.1.2 LY294002

LY294002, a PI3K inhibitor, has shown potential in regulating key processes in RCC. Yue et al. demonstrated that in MUC15-knockdown ACHN and Caki-1 RCC cell lines, treatment with LY294002 for 24 and 48 hours significantly reduced the wound healing rate, reversed the increased cell migration and invasion induced by MUC15 knockdown, and suppressed the upregulated expression of MMP2 and MMP9, the downstream effectors of the PI3K/AKT signaling pathway (216). These findings suggest that LY294002 inhibits RCC cell metastasis by negatively regulating the PI3K/AKT signaling pathway. Furthermore, studies by Hwang et al. revealed that treating RCC cell lines (SN12C and Caki-1) with 20 μM LY294002 for 24 hours significantly reduced intracellular expression of p-AKT and p-S6. Concomitantly, the transcription factor TFE3 accumulated in the nucleus, accompanied by a marked increase in its total protein expression. In addition, expression of immune checkpoint molecules such as PD-L1 and B7-H3 was significantly upregulated, with their levels positively correlating with TFE3 expression (13). These results indicate that LY294002 abrogates TFE3 transcriptional repression mediated by the PI3K/AKT signaling pathway via inhibiting the pathway, thereby promoting TFE3 nuclear translocation and activation. This in turn, regulates expression of immune checkpoint molecules and remodels the tumor microenvironment to enhance anti-tumor immunity. Consequently, combining LY294002 with immune checkpoint inhibitors may enhance therapeutic efficacy via synergy. Thus, LY294002 emerges as a promising candidate for RCC treatment, particularly showing potential for translational research and clinical development in combination immunotherapy strategies.

##### 7.1.1.3 SF1126

SF1126 is a novel prodrug derived from LY294002 via conjugation modification with the RGDS targeting peptide. Through RGDS mediated targeted modification, SF1126 improves the pharmacokinetic deficiencies of LY294002, enabling efficient targeting of tumor tissues and exhibiting favorable water solubility and safety profiles. Further preclinical studies confirmed that SF1126 exerts significant anti-tumor activity by directly inhibiting the PI3K AKT signaling pathway and anti-angiogenic activity by suppressing the HIF-1α/VEGF pathway in various tumor models (217, 218). These findings provide a critical rationale for advancing SF1126 into phase I clinical trials as a pan PI3K inhibitor. Mahadevan et al. conducted a phase I pharmacokinetic and pharmacodynamic study demonstrating that intravenous infusion of SF1126 (840 mg/m²) in patients with mRCC achieved stable disease (SD), with SD persisting for 84 weeks. Notably, all enrolled patients exhibited resistance to prior mTORC1 inhibitor therapy (**Table 2**) (219). These findings indicate that SF1126 shows favorable tolerability and sustained disease control in patients with mRCC, and may offer specific clinical value, particularly for those resistant to mTOR inhibitors. Therefore, further verification of the efficacy and safety of SF1126 as a potential RCC therapeutic candidate in phase II clinical trials is warranted.

##### 7.1.1.4 TGX221

TGX221, a selective PI3Kβ inhibitor, exhibits high specificity for VHL- and SETD2-mutated ccRCC cells and potently suppresses tumor growth and progression (220). Similarly, TGX211 exerts targeting effects on mutations in PTEN and CDKN2A, which are closely associated with alterations in the signaling axes critical for ccRCC cell survival. Despite the promising clinical potential of this class of inhibitors, their translation into clinical practice has not yet been realized to date due to limited efficacy and significant toxicity. This is likely attributable to the extensive signal crosstalk and intricate feedback regulatory networks within the PI3K pathway, which render sustained anti-tumor effects difficult to achieve with single-target inhibition.

#### 7.1.2 AKT inhibitors

AKT inhibitors are classified into two categories based on their chemical structure and mechanism of action: ATP-competitive inhibitors and allosteric inhibitors. ATP-competitive inhibitors bind to the ATP-binding site in the kinase domain of AKT’s active conformation, inhibiting AKT activation. Typical examples include Ipatasertib (GDC-0068), Uprosertib (GSK2141795), Capivasertib (AZD5363), and Afuresertib (GSK2110183), of which only Capivasertib (AZD5363) has received FDA approval for cancer therapy (221–223). Allosteric inhibitors, such as MK-2206, Perifosine, SC66, and Vevorisertib, block AKT phosphorylation and activation by preventing the localization of AKT and its PH domain to the plasma membrane. However, preclinical studies suggest that AKT inhibitors such as GDC-0068 and MK-2206 may be particularly effective for targeting tumors with PIK3CA mutations or loss of PTEN function (224). Both allosteric AKT inhibitors (e.g., Perifosine, MK-2206, SC66) and ATP-competitive inhibitors (e.g., AZD5363) are being tested in clinical trials for ccRCC.

##### 7.1.2.1 Perifosine

Preclinical studies have demonstrated that Perifosine effectively inhibits AKT and S6 ribosomal protein phosphorylation, inducing apoptosis in RCC cell lines *in vitro*. Two independent phase II clinical trials, Perifosine 228 and Perifosine 231, evaluated the efficacy and safety of oral perifosine (100 mg daily) in patients with advanced RCC after failing VEGF-targeted therapy (**Table 2**) (225). In the Perifosine 228 trial, results showed an ORR of 4%, a SD rate of 46%, a median progression-free survival (PFS) of 14.2 weeks (95% CI: 7.7-21.6 weeks), and a 12-week progression-free survival rate of 46%. For the Perifosine 231 trial, the overall ORR was 10% and the SD rate was 32%, with a median PFS of 14 weeks (95% CI: 12.9-20.7 weeks). In Arm A and Arm B of this trial, the median PFS was 14.1 weeks and 14 weeks, respectively, and the 12-week progression-free survival rates were 41% and 44%, respectively. A pooled analysis of these two trials indicated that Perifosine monotherapy demonstrated an overall ORR of 8.1% and a median PFS of 14 weeks, with favorable tolerability and an extremely low incidence of grade 3–4 AEs. Common toxicities primarily included grade 1–2 gastrointestinal reactions such as nausea and diarrhea, musculoskeletal pain, and fatigue. Although these findings suggest that Perifosine exerts modest single agent anti-tumor activity in patients with advanced RCC after failing VEGF targeted therapy, its efficacy did not significantly surpass that of existing second line treatment regimens. Thus, the clinical value of further developing Perifosine as a monotherapy appears limited. Notably,. Zhou et al. discovered that GLI1 and GLI2 are overexpressed in ccRCC and activated by the PI3K/AKT signaling pathway. Further investigations revealed that combining the GLI inhibitor Gant61 with Perifosine significantly suppresses RCC cell proliferation and induces apoptosis in both *in vitro* and *in vivo* models (226). These findings indicate that although Perifosine has limited efficacy as a monotherapy, its favorable tolerability and unique mechanism of action suggest that exploring combination strategies with agents such as mTOR inhibitors may offer greater potential for its future clinical application.

##### 7.1.2.2 MK-2206

MK-2206 is a selective allosteric AKT inhibitor that equipotently inhibits AKT1 and AKT2 isoforms, and its anti-proliferative activity has been demonstrated in preclinical tumor cell line models. Based on these findings, a randomized phase II clinical trial compared the efficacy and safety of MK-2206 to the mTOR inhibitor Everolimus in patients with advanced RCC resistant to VEGF targeted therapy (**Table 2**) (227). Patients received either oral MK-2206–200 mg weekly or oral Everolimus 10 mg daily. Efficacy analysis showed the median PFS in the MK-2206 group was 3.68 months (95% CI: 1.77-5.75), shorter than the 5.98 months (95% CI: 5.03-not reached) in the Everolimus group. Regarding response rates, the MK-2206 group exhibited a bimodal distribution, with 1 patient achieving complete response (CR), 3 achieving partial response (PR), and an ORR of 13.8%. In contrast, no CR or PR occurred in the Everolimus group, and the SD rate was 78.6%. Regarding safety, the most common AEs in the MK-2206 group were maculopapular rash (79.3%), hyperglycemia (69%), and fatigue (62.1%), with grade 3–4 rash and hyperglycemia occurring in 27.6% and 24.1% of patients, respectively. In the Everolimus group, fatigue (78.6%) and hyperlipidemia (64.3%) were the predominant AEs, and severe AEs were relatively infrequent. Additionally, a higher proportion of patients in the MK-2206 group required dose adjustments due to AEs (37.9% vs 21.4%). Collectively, in RCC patients resistant to VEGF targeted therapy, MK-2206 failed to show superior efficacy to Everolimus and was associated with more prominent rash and hyperglycemia toxicities, more frequent dose adjustments, and a higher risk of disease progression. Nevertheless, the bimodal treatment response observed with MK-2206 suggests that AKT inhibitors may have potential efficacy in a specific patient subgroup, offering a research direction for biomarker guided patient selection in future clinical practice.

##### 7.1.2.3 SC66

SC66 is a novel allosteric AKT inhibitor that exerts dual AKT inhibition by inducing AKT ubiquitination and disrupting PH domain binding to PIP3. Studies by Xu et al. demonstrated that SC66 significantly suppresses the viability, proliferation, migration, and invasion of RCC cell lines (786-O, A498) and patient derived primary RCC cells in a dose and time dependent manner, without significant toxicity to normal renal tubular epithelial cells (HK 2, Ren Epi) (228). Mechanistic investigations revealed that SC66 potently inhibits AKT/mTOR signaling pathway activation in RCC cells, as evidenced by reduced p-AKT and p-S6K1 levels, and blocks cell survival signals. However, SC66 retains cytotoxicity in RCC cells with AKT gene silencing or knockout, suggesting an AKT independent anti-tumor mechanism exists for this agent. Further studies showed that SC66 induces cell apoptosis by promoting ROS generation, inhibiting sphingosine kinase 1 (SphK1) activity, facilitating ceramide accumulation, and activating the JNK pathway. This AKT independent effect can be reversed by ROS scavengers (NAC), JNK inhibitors (JNKi), and ceramide antagonists (S1P). *In vivo* studies demonstrated that oral SC66 administration (10 or 25 mg/kg) significantly inhibited subcutaneous 786-O cell xenograft growth in SCID mice without causing obvious body weight changes. Analysis of tumor tissues revealed AKT/mTOR signaling pathway inhibition, reduced SphK1 activity, ceramide accumulation, and JNK activation, consistent with *in vitro* observed mechanisms. These findings suggest that SC66 suppresses RCC progression through both AKT dependent and AKT independent mechanisms, exerts significant anti-tumor activity *in vitro* and *in vivo*, and exhibits low cytotoxicity to normal cells, providing a novel strategy for RCC treatment. Therefore, further preclinical safety evaluations and clinical trials are needed to validate its clinical translation potential.

##### 7.1.2.4 Capivasertib (AZD5363)

Capivasertib (AZD5363) is the first AKT inhibitor approved by the FDA in November 2023, approved for treating adult patients with metastatic breast cancer who are hormone receptor positive, human epidermal growth factor receptor 2 negative, and harbor at least one genetic alteration in PIK3CA, AKT1, or PTEN (229). Beyond breast cancer, Capivasertib has shown initial promising anti-tumor potential in early phase clinical trials for other tumor types. For instance, in subprotocol EAY131-Y of the National Cancer Institute Molecular Analysis for Therapy Choice (NCI-MATCH) trial, results showed Capivasertib exerted clinically meaningful anti-tumor activity in patients with the AKT1 E17K mutation, particularly in refractory malignant tumors (230). In addition, another NCI-MATCH trial study further confirmed Capivasertib achieved an ORR of approximately 20% in patients with AKT E17 mutations, further validating its therapeutic value for AKT mutant tumors (231). However, unlike research progress in breast cancer and other tumor types, Capivasertib application in RCC remains limited to preclinical and early clinical exploration stages. Kadomoto S et al. demonstrated that AZD5363 effectively blocks tumor associated macrophage induced migration and epithelial mesenchymal transition in RCC cells by inhibiting AKT activity, suggesting its therapeutic potential for RCC (232). Nevertheless, the incidence of the AKT1 E17K mutation is relatively low in RCC, particularly in ccRCC, and a definitive link between this mutation and AKT inhibitor sensitivity remains unestablished. Therefore, future clinical prospects for Capivasertib in RCC will likely depend on biomarker based precise screening and rational combination therapy development. For example, such strategies may involve combining Capivasertib with mTOR inhibitors to counteract feedback activation, or with immune checkpoint inhibitors to remodel the tumor microenvironment.

#### 7.1.3 mTOR inhibitors

The mTOR inhibitors are the most extensively studied targeted therapies for the PI3K-AKT-mTOR signaling pathway and were the first to enter clinical practice (233). Rapamycin and its derivatives (rapalogs) are first-generation mTOR inhibitors, including Sirolimus, Everolimus (RAD001) and Temsirolimus (CCI-779). These inhibitors bind to FKBP12, suppressing mTORC1 activity. Based on findings from the Multicenter International LAM Efficacy trial, Sirolimus was approved as the first and only treatment for lymphangioleiomyomatosis (234). Temsirolimus, the first mTORC1 inhibitor to receive FDA approval, was approved in 2007 for the treatment of advanced RCC (233, 235); Everolimus was approved by the FDA in 2009 for the treatment of patients with advanced RCC who have failed treatment with Sunitinib or Sorafenib (236). Both Temsirolimus and Everolimus are recommended as first-line treatments for ccRCC patients with poor prognosis or those who have failed TKI therapy. However, as rapalogs specifically target mTORC1, their use may lead to the reactivation of upstream PI3K/AKT signaling via feedback loops (237), prompting the development of second and third-generation mTOR inhibitors.

Second-generation mTOR inhibitors are ATP-competitive, highly selective mTOR inhibitors capable of simultaneously inhibiting both mTORC1 and mTORC2. These inhibitors are currently undergoing clinical trials for cancer therapy and include OSI-027 (OSI), Vistuserib (AZD2014), AZD8055, and Sapanisertib (TAK-228) (11). Among them, Vistuserib (AZD2014) and Sapanisertib (TAK-228) have been used in the research of ccRCC. The dual mTORC1/2 inhibitor AZD2014 significantly reduced the survival and growth of RCC cells (786-O and A498 cell lines) in both *in vitro* and *in vivo* models, exhibiting higher efficacy than conventional mTORC1 inhibitors. However, AZD2014 induced protective autophagy in RCC cells (238). An open-label, multicenter, randomized phase II trial evaluated the efficacy and safety of AZD2014 compared with Everolimus in patients with metastatic ccRCC after failing VEGF-targeted therapy (**Table 2**) (239). Results showed the median PFS in the AZD2014 group (50 mg orally twice daily) was 1.8 months, significantly shorter than the 4.6 months in the Everolimus group (10 mg orally daily; HR = 2.8, 95% CI: 1.2-6.5). As AZD2014 failed to meet efficacy expectations, the trial was prematurely terminated in June 2014. At termination, the median OS in the AZD2014 group was 6.2 months, significantly lower than the 16.7 months in the Everolimus group (HR = 3.1, 95% CI: 1.1-8.4). Grade 3–4 AEs incidences were 35% and 48% in the two groups, respectively, with no statistically significant difference. Although AZD2014 demonstrated acceptable safety and pharmacokinetic profiles, it was significantly inferior to Everolimus for both PFS and OS. Thus, this study does not support further clinical investigation of AZD2014 in patients with VEGF-refractory metastatic ccRCC. Sapanisertib (TAK-228, formerly MLN0128/INK128) is another dual mTORC1/2 inhibitor with high oral bioavailability and selectivity. A preclinical study by Ingels et al. demonstrated that MLN0128 (INK128) effectively inhibits primary RCC growth and reduces liver metastases from RCC (240). Moreover, in terms of inhibiting primary RCC growth and metastasis, MLN0128 outperformed Temsirolimus, positioning it as a promising anti-RCC agent. A phase I trial demonstrated that Sapanisertib exhibited manageable safety and preliminary anti-tumor activity in patients with RCC. Notably, in the subgroup of patients naive to prior mTORC1 inhibitor therapy, the ORR reached 22%, significantly superior to that of conventional rapalogs (ORR: 1%-8.6%), with the longest partial response duration exceeding 16 months (241). A subsequent randomized phase II trial evaluated the efficacy and safety of Sapanisertib monotherapy (30 mg weekly), the combination of Sapanisertib (4 mg daily for 3 consecutive days/week) plus the PI3Kα inhibitor TAK-117 (200 mg daily), and Everolimus monotherapy (10 mg daily) in patients with advanced ccRCC after failing VEGF-targeted therapy (**Table 2**). Results showed no statistically significant differences in median PFS or OS among the three groups. Discontinuation rates due to treatment emergent AEs were higher in both the Sapanisertib monotherapy group (28.1%) and the combination group (29.0%) than in the Everolimus group (15.6%) (242). Collectively, these findings indicate that in patients with VEGF-refractory advanced ccRCC, neither Sapanisertib monotherapy nor its combination with TAK-117 demonstrated superior efficacy to Everolimus, and both were associated with less favorable tolerability. This suggests that simple blockade of the mTORC1/2 signaling pathway may be insufficient to reverse VEGF inhibitor resistance in RCC, warranting exploration of more precise pathway modulation strategies or combination regimens in the future.

RapaLinks are third-generation mTOR inhibitors that selectively inhibit mTORC1 (243). RapaLinks can deepen mTORC1 inhibition and address resistance mechanisms associated with first- and second-generation mTOR inhibitors by targeting both the variant FRB structural domain and the orthosteric catalytic domain of mTOR. Rapalink-1 is a drug that combines rapamycin and the mTOR kinase inhibitor MLN0128. Kuroshima et al. found that Rapalink-1 had a more pronounced effect on proliferation, migration and invasion of RCC cells compared to Temsirolimus in the treatment of Sunitinib-sensitive and resistant RCC cells (244). Furthermore, Rapalink-1 could significantly inhibit protein phosphorylation in the PI3K-AKT-mTOR signaling pathway, while Temsirolimus could not. RMC-4627 is a potent, highly selective dual site mTORC1 inhibitor. Preclinical studies demonstrated that it effectively inhibited phosphorylation of mTORC1 downstream substrates 4E-BP1 and S6 at nanomolar concentrations (0.3–10 nM), with no significant effect on AKT S473 phosphorylation mediated by mTORC2, demonstrating 13 to 18 fold selectivity for mTORC1 (245). Compared with conventional mTOR kinase inhibitors such as MLN0128, RMC-4627 demonstrates greater inhibitory potency and more sustained target suppression. Furthermore, as a dual site mTORC1 inhibitor, RMC-5552 became the first drug candidate in this class to enter clinical development due to its favorable selectivity, stability, and pharmacodynamic properties, advancing to the phase I/IB clinical trial stage for treating solid tumors with mTORC1 activation (246). This progress offers a novel therapeutic option for cancer patients with PI3K/mTOR pathway aberrations or KRAS mutations.

#### 7.1.4 Dual PI3K/mTOR inhibitors

mTOR and PI3K are part of the same PIKK superfamily, sharing similar structural compositions and activation mechanisms (247). Dual PI3K/mTOR inhibitors can fully block the aberrant activation of the PI3K-AKT-mTOR signaling pathway, preventing the feedback activation of the PI3K/AKT pathway. Thus, targeting key points of the same pathway may lead to better anti-cancer activity and also overcome resistance to single inhibitor therapy. Several clinical trials are currently investigating dual PI3K/mTOR inhibitors, including Dactolisib (BEZ235), Apitolisib (GDC-0980), PKI-587, SF1126, Bimiralisib (PQR309), Paxalisib (GDC-0084), Voxtalisib (SAR245409, XL765), and LY3023414. Furthermore, preclinical studies have demonstrated superior activity of dual PI3K/mTOR inhibitors compared to mTORC1-targeted rapalogs. Several of these dual PI3K/mTOR inhibitors are in preclinical and clinical trials for RCC treatment, such as BEZ235, Apitolisib (GDC-0980), GNE-477, VS-5584, and SN202.

##### 7.1.4.1 BEZ235

BEZ235 in RCC cell lines has shown more effective cell growth arrest and anti-tumor activity *in vitro* and *in vivo* than mTORC1 inhibition alone (248). A single-center phase IB trial evaluated the safety and maximum tolerated dose of BEZ235 in patients with advanced RCC (**Table 2**). Ten patients with advanced RCC were enrolled and received oral BEZ235 at doses of 200, 300, or 400 mg twice daily (249). Results showed that dose-limiting toxicities occurred at all BEZ235 dose levels, including fatigue, rash, nausea, vomiting, diarrhea, and mucositis. Among them, 50% of patients experienced grade 3–4 TEAEs, and 50% withdrew from the study due to toxicities. Collectively, these findings indicate that BEZ235 exhibits a poor tolerability profile and significant toxicities in patients with advanced RCC, with no demonstrable anti-tumor activity. Therefore, further clinical development of BEZ235 in its current formulation is not warranted.

##### 7.1.4.2 Apitolisib (GDC-0980)

Oral Apitolisib (GDC-0980) has shown favorable pharmacokinetics and bioactivity in a phase I trial in patients with advanced solid tumors (250). A phase I trial of Apitolisib demonstrated that the 40 mg dose had preliminary activity and was selected as the recommended phase II dose. A subsequent randomized open-label phase II trial compared the efficacy and safety of Apitolisib (40 mg daily) with Everolimus (10 mg daily) in patients with mRCC after failing VEGF-targeted therapy (**Table 2**) (251). Results showed the median PFS in the Apitolisib group was 3.7 months, significantly shorter than the 6.1 months in the Everolimus group (HR = 2.12), with no advantage observed across subgroups. Overall survival also favored Everolimus (16.5 vs. 22.8 months; HR = 1.77). No statistically significant difference in ORR was observed between groups (7.1% vs. 11.6%). In terms of safety, the Apitolisib group had a higher incidence of grade 3–4 AEs (74% vs. 44%), primarily hyperglycemia and rash, leading to more discontinuations due to AEs. Biomarker analysis revealed that VHL gene mutations were associated with Everolimus efficacy but not with that of Apitolisib, while high HIF-1α expression correlated with improved PFS in patients in both groups. Collectively, in patients with mRCC after failing VEGF-targeted therapy, Apitolisib’s dual-target inhibition strategy did not demonstrate superior efficacy to Everolimus, a single-target mTOR inhibitor. Excessive pathway inhibition by Apitolisib may induce severe toxicities, thereby limiting therapeutic dose and treatment duration. These findings suggest that single-target mTORC1 inhibition remains the optimal option for this population, and VHL and HIF-1α may serve as potential predictive biomarkers for mTOR inhibitor efficacy, although their clinical utility requires further validation in prospective studies.

##### 7.1.4.3 GNE-477

GNE-477 is a novel dual PI3K/mTOR inhibitor that acts by significantly inhibiting phosphorylation of p85, AKT (Ser473/Thr308), p70S6K1, and S6, without affecting ERK1/2 phosphorylation levels. Moreover, GNE-477 shows greater anti-tumor activity than other inhibitors (LY294002, Perifosine, and AZD2014). In primary human RCC cells, GNE-477 significantly suppresses cell viability, proliferation, colony formation, and cell cycle progression in a dose and time dependent manner, impairs cell migration and invasion, and exhibits no significant cytotoxicity to normal renal epithelial cells (HK-2). In a nude mouse xenograft model, intraperitoneal GNE-477 injection (10 or 50 mg/kg) significantly inhibited tumor volume and weight, was more efficacious than the mTOR inhibitor AZD2014 at equivalent doses, and caused no significant body weight loss or toxic reactions (252). Collectively, GNE-477 demonstrates potent anti-RCC activity both *in vitro* and *in vivo* via specific blockade of the PI3K-Akt-mTOR signaling pathway, providing a novel drug candidate for molecularly targeted RCC therapy.

##### 7.1.4.4 VS-5584

VS-5584 has shown potent inhibitory effects on the PI3K-AKT-mTORC1/2 signaling pathway in RCC 786-O cell line and primary human RCC cells, leading to reduced cell survival, proliferation, cell cycle progression, and migration. In a xenograft mouse model of RCC, VS-5584 effectively suppressed tumor growth with good tolerability and no significant toxicity observed. Notably, BRD4 was identified as a key resistance factor to VS-5584; inhibiting BRD4 enhanced the compound’s anti-tumor activity, suggesting its potential as a therapeutic candidate for RCC treatment (253).

##### 7.1.4.5 SN202

As an ATP-competitive dual PI3K/mTOR inhibitor, SN202 potently inhibits PI3Kα (IC_50_=3.2 nM), PI3Kγ (IC_50_=3.3 nM), and mTOR (IC_50_=1.2 nM), with significantly greater inhibitory activity than the tool compound PI-103. In 786-O, A498, and ACHN renal cancer cell lines, SN202 dose-dependently suppressed cell proliferation, with IC_50_ values of 0.486 μM, 0.176 μM, and 0.298 μM, respectively, and the strongest inhibition observed in the 786-O cell line. *In vivo*, oral SN202 administration (5 and 20 mg/kg) significantly inhibited 786-O xenograft growth in nude mice, achieving tumor weight inhibition rates of 68.61% and 88.99%, respectively, without obvious body weight loss or toxic reactions (254). Collectively, SN202 demonstrates significant *in vitro* and *in vivo* anti-RCC activity with favorable safety and pharmacokinetic profiles, holding promise as a drug candidate for RCC treatment.

### 7.2 Development of natural products for PI3K-AKT-mTOR in RCC

Natural compounds derived from plants and animals have exhibited remarkable anti-cancer and preventive properties, particularly in the context of RCC. Herbal medicines offer multiple advantages, such as improving chemotherapy and targeted treatments, reducing the risk of metastasis and recurrence, extending patient survival, and enhancing overall quality of life. Research has consistently highlighted the therapeutic potential of natural products and their derivatives, especially in combination with established treatments or when addressing drug resistance. These compounds have demonstrated the ability to inhibit RCC cell growth, trigger apoptosis, promote autophagy, and exert anti-angiogenic effects. Natural products with diverse biological activities, such as Sinomenine, Resveratrol, Hemsleya amabilis Diels, Brusatol, and Gypenosides, may serve as potential candidates for the treatment of advanced RCC.

#### 7.2.1 Sinomenine

Sinomenine, an isoquinoline alkaloid derived from the Chinese herb Cymbidium, exhibits notable anti-inflammatory, anti-rheumatic, analgesic, and immunomodulatory effects. Sinomenine dose-dependently inhibited cell viability in the human RCC ACHN cell line at concentrations of 0.5, 1, and 3 mM, significantly impaired its sphere-forming ability, and downregulated the expression of proliferation markers PCNA and Ki67. This compound also promoted cell apoptosis by upregulating cleaved caspase-3 and caspase-9. In addition, Sinomenine modulated autophagy-related proteins: it upregulated Beclin1 and the LC3-II/LC3-I ratio while downregulating p62, thereby inducing autophagy. Further studies demonstrated that Sinomenine exerted its core anti-tumor effect through inhibition of the PI3K-AKT-mTOR signaling pathway, manifested by downregulation of p-PI3K/PI3K, p-AKT/AKT, and mTOR expression, reduced AKT membrane translocation, and consequent suppression of pathway activity (255). These findings indicated that Sinomenine exerts anti-RCC effects *in vitro* models through targeted inhibition of the PI3K-AKT-mTOR pathway, inducing cellular autophagy and apoptosis. However, the anti-tumor activity and safety of this compound remain unverified in animal models; further *in vivo* experiments are required to enable in-depth evaluation and clarify its translational potential.

#### 7.2.2 Resveratrol

Resveratrol (RES), a natural polyphenolic compound naturally occurring in berries, peanuts, and plant roots such as Veratrum album and Polygonum cuspidatum, exhibits chemopreventive properties and anti-tumor activity (256, 257). Studies demonstrated that RES inhibits RCC cell viability in a concentration and time dependent manner, with IC_50_ of 132.9 ± 1.064 μM and 112.8 ± 1.191 μM in ACHN and A498 cell lines, respectively. Notably, RES shows no significant cytotoxicity in normal renal tubular epithelial cells (HK-2) but induces lactate dehydrogenase release in RCC cells, suggesting selective cytotoxicity against RCC cells (258). Further mechanistic investigations revealed that RES suppresses RCC cell proliferation, migration, and invasion by inhibiting AKT and ERK1/2 signaling pathways and downregulating epithelial-mesenchymal transition (EMT)-related protein and MMPs expression. However, *in vivo* use of free RES is limited by poor stability and low bioavailability. Recent studies showed that liposomal encapsulation improves its physicochemical properties. Resveratrol liposomes (RES-Lips) facilitate sustained drug release, enhance cellular uptake, and scavenge free radicals to mitigate oxidative damage (259). Notably, RES-Lips are considered synergistic agents in combination therapies. Wang et al. found that RES-Lips (16–32 mmol/L) synergized with Sorafenib (23.1 μmol/L) to inhibit 786-O/S cell viability, reverse Sorafenib resistance (reversal rate=0.58), and induce G1/S phase arrest. In a murine RCC xenograft model, intragastric dosing of RES-Lips (200 μmol/kg/day) combined with Sorafenib (20 mg/kg/day) for 14 days resulted in a tumor growth inhibition rate of 90.1%, with 50% of mice showing CR and 100% survival, significantly outperforming monotherapy. Mechanistically, this combination enhanced drug resistant RCC sensitivity to Sorafenib and reversed resistance via concurrent inhibition of PI3K-AKT-mTOR and VHL-HIF signaling pathways (260). Collectively, as a chemosensitizer, RES-Lips synergistically enhances anti-RCC efficacy and reverses Sorafenib resistance by targeting PI3K-AKT-mTOR and VHL-HIF pathways, demonstrating promising therapeutic potential. Nevertheless, its clinical translational value requires further investigation to validate.

#### 7.2.3 Hemsleya amabilis Diels

Hemsleya amabilis Diels (HRE), also known as ‘xue dan’ or Chinese small serpentine, belongs to the Cucurbitaceae family. Modern pharmacological studies have shown that HRE possesses anti-tumor and anti-inflammatory properties. Ultra-high performance liquid chromatography-mass spectrometry analysis identified 739 compounds in HRE, comprising flavonoids (22.6%), alkaloids (17.5%), and terpenoids (11.7%). Among these, epitulipinolide diepoxide showed stable binding affinity for PIK3CA with a binding energy of -7.22 kJ/mol, suggesting its potential inhibitory activity against this target. *In vitro* studies demonstrated that HRE suppressed proliferation of RCC cell lines (RCC4, OS-RC-2, and ACHN) in a dose and time dependent manner; at 20 μg/mL, HRE showed the most pronounced effects by inhibiting proliferation, inducing G2/M cell cycle arrest, and promoting apoptosis. In nude mouse xenograft models, HRE at 150 or 300 mg/kg significantly inhibited tumor growth without obvious histological abnormalities in major organs, with the high dose group exhibiting superior efficacy. RNA sequencing and network pharmacology analyses revealed that HRE anti-RCC activity was closely linked to the PI3K/AKT signaling pathway. IGF-1, a PI3K activator, reversed HRE induced apoptosis and cell cycle arrest, whereas LY294002, a PI3K inhibitor, enhanced HRE anti-tumor effects. Furthermore, HRE upregulated P21 (CDKN1A) expression through the PI3K/AKT pathway, thereby inducing G2/M arrest, and regulated apoptosis related proteins (Bax, Bcl-2, and Caspase family members) and EMT markers (E-cadherin, N-cadherin) (261). Collectively, *in vitro* and *in vivo* studies confirm that HRE exerts anti-RCC effects through targeting the PI3K/AKT pathway to inhibit proliferation, induce cell cycle arrest and apoptosis, with a favorable safety profile. Thus, HRE represents a promising candidate agent for RCC therapy and warrants further development.

#### 7.2.4 Brusatol

Brusatol, an active compound extracted from Bruceajavanica (L.) Merr., exhibits various biological effects, including anti-inflammatory, anti-tumor, and anti-malarial properties. It has demonstrated effective anti-cancer activity, targeting multiple pathways to inhibit tumor growth and showing strong inhibitory effects against many malignancies, including pancreatic, breast, and nasopharyngeal cancers (262–264). Wang et al. demonstrated that Brusatol exerted concentration dependent anti-proliferative, pro-apoptotic, anti-migratory, and anti-invasive effects on RCC lines (A498, ACHN, OS-RC-2) at 50 to 200 nmol/L, with 48 hour IC_50_ of 79.11 nmol/L, 121.4 nmol/L, and 111.2 nmol/L, respectively. Mechanistic studies revealed that Brusatol markedly upregulated PTEN expression, inhibited phosphorylation ratios of p-PI3K/PI3K and p-AKT/AKT, and modulated apoptosis related protein expression (elevated Bax/Bcl-2 ratio) and EMT marker expression (upregulated E-cadherin, downregulated N-cadherin and vimentin). Notably, Brusatol anti-tumor effects were significantly abrogated following PTEN knockout, indicating its action depended on the PTEN-PI3K-AKT signaling pathway. In addition, studies suggested Brusatol might promote PTEN transcriptional expression by enhancing histone acetylation (265). Collectively, Brusatol exerts anti-RCC activity *in vitro* models by modulating the PTEN-PI3K-AKT signaling pathway, providing experimental evidence for its further development as a drug candidate. Nevertheless, its *in vivo* efficacy and clinical translational potential remain to be verified.

#### 7.2.5 Gypenosides

Gypenosides, the main active component of Gynostemma pentaphyllum, a plant from the Cucurbitaceae family, are widely used in traditional medicine to treat tumors and hyperlipidemia. Gypenosides have demonstrated significant anti-cancer activity both *in vitro* and *in vivo*. Liu et al. demonstrated that Gypenosides significantly inhibited the viability of RCC cell lines 786-O and Caki-1, reduced colony formation, and induced apoptosis, with IC_50_ of 180.0 μg/mL and 453.0 μg/mL, respectively. After 24-hour treatment at 200 μg/mL (786-O) or 500 μg/mL (Caki-1), cell apoptosis rates were markedly elevated. Mechanistic investigations revealed that Gypenosides suppressed aberrant activation of the PI3K-AKT-mTOR signaling pathway by downregulating PIK3CA, AKT, and mTOR mRNA expression and reducing p-AKT and p-mTOR protein expression (266). These findings suggested that Gypenosides induce RCC cell apoptosis through targeted modulation of the PI3K-AKT-mTOR pathway. However, this study did not perform *in vivo* animal experiments to verify anti-tumor efficacy, and long-term toxicological data are also lacking. Therefore, whether Gypenosides can serve as a potential candidate for RCC treatment remains unclear, requiring additional experimental evidence in future studies to confirm clinical application potential.

## 8 Resistance mechanisms of the PI3K-AKT-mTOR signaling pathway in RCC

Resistance to targeted therapy represents a major clinical challenge in RCC. While inhibitors of the PI3K-AKT-mTOR signaling pathway have demonstrated certain efficacy in RCC management, widespread drug resistance substantially curtails their clinical benefit. Accumulating evidence underscores that such resistance constitutes a multifactorial, dynamic evolutionary process characterized by adaptive rewiring of tumor cell signaling networks and their coordinated crosstalk with the tumor microenvironment. Based on the timing and mechanisms of resistance development, resistance to targeted therapy can be categorized into two distinct types: primary resistance, defined by pre-existing genetic alterations or clonal heterogeneity that preclude initial response, and secondary resistance, which emerges following an initial therapeutic response due to drug pressure-driven clonal selection and adaptive phenotypic changes (6).

Tumor heterogeneity and clonal evolution represent the fundamental biological basis of targeted therapy resistance in RCC. Such resistance arises either from pre-existing, inhibitor-insensitive clonal subpopulations present before treatment (intrinsic resistance) or from *de novo* genetic alterations emerging under drug selection pressure (acquired resistance). The latter primarily encompasses activating mutations of PIK3CA/AKT1and PTEN deletion, both of which drive the emergence of resistant phenotypes. For example, PIK3CA mutations have been linked to the therapeutic response to Buparlisib. PTEN deletion is strongly associated with diminished sensitivity to Sunitinib and Bevacizumab (267, 268); notably, its expression can be upregulated by the natural product Brusatol, which reverses the resistant phenotype (265). Furthermore, mutations within the FKBP12 domain of the mTOR complex, along with functional abnormalities of TSC1/2 and REDD1, can compromise the efficacy of mTORC1 inhibitors (e.g., Everolimus, Temsirolimus). These molecular alterations are currently under investigation as potential predictive biomarkers for therapeutic efficacy (102). The development of secondary resistance is predominantly involves intra-pathway feedback reactivation​ and inter-pathway compensatory crosstalk. Among these, intra-pathway feedback reactivation stands as a central driver of acquired resistance. For instance, mTORC1 inhibitors relieve negative feedback inhibition of IRS-1, triggering compensatory hyperactivation of upstream PI3K/AKT signaling and subsequently attenuating antitumor efficacy (237). Similarly, inhibition of AKT or PI3K can upregulate or activate upstream RTKs, thereby reactivating downstream signal transduction (269). In RCC, inactivation of the mTORC1/S6K feedback loop not only induces aberrant PI3K/AKT activation but also mediates cross-resistance to VEGF-targeted therapies via transcriptional upregulation of HIF-α (270, 271). Notably, pharmacological inhibition of the PI3K-AKT-mTOR pathway reverses Sunitinib- and Sorafenib-resistant phenotypes driven by aberrant AKT activation, underscoring that this pathway represents a potential therapeutic node to overcome drug resistance (267, 272). Inter-pathway compensatory crosstalk endows tumor cells with critical bypass escape routes. Upon inhibition of the PI3K-AKT-mTOR signaling pathway, alternative survival pathways such as RAS-MAPK/ERK and JAK/STAT can be activated to sustain tumor cell proliferation and viability (273, 274). In ccRCC, the HIF signaling axis driven by VHL inactivation constitutes a parallel regulatory network; its downstream pro-angiogenic and metabolic reprogramming effects overlap extensively with the PI3K pathway, markedly attenuating sensitivity to single-pathway inhibition. Furthermore, cross-activation of compensatory signaling pathways directly counteracts the inhibitory effects of targeted agents. Studies have demonstrated that resistance to mTOR inhibitors is frequently accompanied by increased AKT membrane translocation and rebound phosphorylation of ERK1/2, which partially explains why dual mTORC1/2 inhibitors such as Sapanisertib failed to outperform Everolimus in phase II clinical trials (242). In addition, this pathway indirectly modulates responses to immunotherapy by remodeling the tumor microenvironment, promoting infiltration of tumor-associated macrophages and upregulating immune checkpoint molecules such as PD-L1) (275). Notably, a positive feedback loop operates in ccRCC: VHL inactivation drives HIF accumulation, which in turn activates PI3K/AKT. HIF can directly bind the PIK3CA promoter to enhance its transcription, thereby stabilizing and amplifying the drug-resistant phenotype.

The aforementioned drug resistance mechanisms directly manifest as severe clinical challenges: patients frequently exhibit rapid disease progression, resulting in minimal extension of PFS and OS, alongside suboptimal ORR. To address these limitations and enhance patient outcomes, future research must prioritize multi-target combinatorial intervention strategies. For instance, the use of dual or multiple inhibitors to block intra-pathway feedback activation, combined targeting of parallel signaling pathways, and combination with anti-angiogenic drugs, immune checkpoint inhibitors, etc., to achieve systematic inhibition of the tumor signaling network. In summary, in-depth elucidation of the drug resistance mechanisms of the PI3K-AKT-mTOR signaling pathway and exploration of effective combined therapeutic strategies are key directions to improve the efficacy of targeted therapy for RCC and enhance the long-term prognosis of patients. In conclusion, in-depth elucidation of PI3K-AKT-mTOR pathway-mediated drug resistance mechanisms and exploration of effective combinatorial therapeutic strategies represent critical avenues to enhance the efficacy of targeted therapy for RCC and improve long-term patient outcomes.

## 9 Conclusions and prospects

RCC is the second most common malignant tumor of the urinary system, often presenting with an insidious onset. Approximately 30% of patients have distant metastasis at initial diagnosis. Among RCC subtypes, ccRCC constitutes the predominant form, demonstrating the highest malignant potential marked by high vascularity and metabolic reprogramming, as well as a propensity for rapid progression and metastasis. Aberrant activation of the PI3K-AKT-mTOR signaling pathway plays a central role in RCC development and progression, participating in key pathological hallmarks including genetic alterations, angiogenesis, metabolic reprogramming, apoptotic imbalance, autophagic dysfunction, and drug resistance. Furthermore, this pathway cross-talks extensively with other signaling cascades and is subject to regulation by epigenetic mechanisms such as miRNAs, thereby forming a multidimensional regulatory network. Consequently, the PI3K-AKT-mTOR pathway is recognized as a critical therapeutic target for precision intervention in RCC. However, despite preclinical studies revealing the complexity of this pathway’s regulatory network, its clinical translation has lagged significantly behind expectations. To date, only two mTORC1 inhibitors, Temsirolimus and Everolimus, have received FDA approval for advanced RCC. Numerous small-molecule inhibitors and natural products targeting this pathway have demonstrated promising antitumor activity in preclinical models, yet their development has stalled in subsequent clinical translation due to limitations including modest efficacy, significant toxicity, feedback reactivation of signaling pathways, and drug resistance. This underscores that future targeted therapy for RCC must shift from a “pan-inhibition” paradigm to a “precision intervention” strategy.

Based on existing evidence and unmet clinical needs, future research on targeted therapy for RCC must prioritize the following direction: (1) Developing a biomarker-driven system for precise stratification and personalized treatment. Current studies confirm that PIK3CA mutations, PTEN deletions, TSC1/2 mutations, and high HIF-2α expression are associated with predictive value for sensitivity to PI3K-AKT-mTOR pathway inhibitors. Biomarkers such as elevated fasting plasma glucose levels and VHL mutation status have also demonstrated preliminary predictive utility in clinical trials. However, the clinical translation of these candidate biomarkers remains incomplete, mainly limited by insufficient detection sensitivity caused by low−frequency mutations and intratumoral heterogeneity, the lack of standardized cutoff definitions, and a predominance of retrospective studies with scarce prospective validation evidence. In the future, dynamic molecular stratification strategies based on circulating tumor DNA should be established using liquid biopsy techniques to real−time monitor pathway mutation status and the evolution of drug−resistant clones. Multidimensional predictive models should also be constructed by incorporating radiomic features. Meanwhile, umbrella and basket trial designs should be adopted to prospectively validate the improvement in survival benefits conferred by biomarker−guided treatment decisions. This will accelerate the translation of biomarkers from the laboratory to clinical practice and provide a reliable basis for precise stratification and the formulation of personalized therapeutic strategies for RCC patients. (2) Promoting multi-omics integration and dissection of spatial metabolic heterogeneity. The introduction of single-cell and spatially resolved omics technologies is crucial for reconstructing our understanding of PI3K-AKT-mTOR pathway regulation. Single-cell RNA sequencing can reveal intratumoral heterogeneity in RCC at the single-cell level and identify pathway activation patterns and dynamic evolutionary trajectories of distinct clonal subpopulations. Spatial transcriptomics can localize pathway activation status *in situ*, delineating the spatial co-localization network of metabolic reprogramming, angiogenesis, and immune exclusion. The integration of this technological system is expected to advance RCC research from qualitative judgments of “whether the pathway is activated” to quantitative dissection of “in which cell type and microenvironment the pathway is activated”, providing a new dimension for screening combination therapeutic targets. (3) Exploring novel targeted drugs and innovative technological pathways. Beyond the optimization of existing inhibitors, future efforts should focus on developing breakthrough therapeutic strategies. First, deepen the exploration of novel therapeutic agents such as allosteric inhibitors and bispecific antibodies to broaden the spectrum of targeted interventions. Second, advance the development of PROTAC technology to achieve selective degradation of mTOR or AKT proteins, thereby circumventing feedback activation at the source. Third, strengthen translational research on natural products and their derivatives, systematically elucidating their mechanisms of action, pharmacokinetic profiles, and clinical translational feasibility, with particular attention to their potential value as sensitizers in combination therapy. These approaches will provide more selective and durable intervention tools for targeted therapy of RCC. (4) Developing resistance-reversal strategies centered on combination therapy. The complex regulatory characteristics of the PI3K-AKT-mTOR pathway, including multilayer feedback activation, bypass signaling compensation, and metabolism-signaling coupling, determine that single-node inhibition is difficult to achieve durable efficacy. Future combination therapeutic strategies should be mechanism-oriented, focusing on three major approaches: dual/multi-target synergistic inhibition, simultaneous blockade of parallel signaling axes, and cross-therapy mode combinations. It should be particularly emphasized that the design of combination regimens should avoid empirical drug combinations. Instead, combinations should be optimized based on the molecular characteristics of drug-resistant clones to maximize the risk of toxicity overlap while achieving synergistic efficacy. (5) Promoting the deep integration of artificial intelligence and precision medicine. Integrating multi-omics data and clinical phenotypic information through machine learning algorithms to construct predictive models for PI3K-AKT-mTOR pathway activity will provide new tools for patient stratification, dynamic monitoring of therapeutic response, and early warning of drug resistance in RCC. Such models will offer technical support for adaptive clinical trials with “real-time treatment adjustment”, driving the transition of precision medicine from a static stratification model to a dynamic decision-making paradigm.

In summary, the core competitiveness of targeted therapy for RCC will no longer depend solely on the development of novel highly specific inhibitors, but more on the ability to establish a trinity precision medicine paradigm: molecular typing-dynamic monitoring-mechanism-driven combination therapy. Through the systematic integration of biomarker discovery, cutting-edge multi-omics technologies, functional models, and intelligent clinical trial design, it is promising to break through the long-standing bottlenecks in pathway-targeted therapy and ultimately achieve substantial improvements in the efficacy and prognosis of patients with RCC.

## Glossary

RCC: renal cell carcinoma
OS: overall survival
TME: tumor microenvironment
VHL: von Hippel-Lindau
PTEN: phosphatase and tensin homolog
HIF: hypoxia-inducible factor
VEGF: vascular endothelial growth factor
PDGF: platelet-derived growth factor
EGF: epidermal growth factor
RTKs: receptor tyrosine kinases
TCGA: the Cancer Genome Atlas
mRCC: metastatic RCC
FDA: the US Food and Drug Administration
TKIs: tyrosine kinase inhibitors
FKBP12: FK506-binding protein-12
ICIs: immune checkpoint inhibitors
EAU: European Association of Urology
GPCRs: G protein-coupled receptors
PKB: Protein kinase B
ULK1: unc-51-like kinase 1
SGK: serum/glucocorticoid-regulated kinase
RHEB: ras homolog enriched in the brain
TSC1/2: tuberous sclerosis complex ½
IGF: insulin-like growth factor
IRS: insulin receptor substrate
PIP2: phosphatidylinositol-4,5-bisphosphate
PIP3: phosphatidylinositol-3,4,5-trisphosphate
PH: pleckstrin homology
4E-BP1: eIF4E-binding protein 1
p70S6K: p70 ribosomal protein S6 kinase
TCA: tricarboxylic acid
GLUT-1: glucose transporter-1
HK: hexokinase
PDH: pyruvate dehydrogenase
PPP: pentose phosphate pathway
NADPH: nicotinamide adenine dinucleotide phosphate
ROS: reactive oxygen species
G6PD: glucose-6-phosphate dehydrogenase
CEs: cholesteryl esters
FASN: fatty acid synthase
CPT1A: carnitine palmitoyltransferase 1A
ACLY: ATP citrate lyase
ACC: acetyl-CoA carboxylase
SCD1: stearoyl-CoA desaturase 1
AMPK: AMP-activated protein kinase
GSH: Glutathione
α-KG: α-ketoglutarate
GLUD1: glutamate dehydrogenase 1
ASS1: arginine succinate synthase 1
IDO1: indoleamine 2,3-dioxygenase 1
LTB4R: leukotriene B4 receptor
miRNAs: microRNAs
CLL: chronic lymphocytic leukemia
NHL: B-cell non-Hodgkin’s lymphoma
SLL: small lymphocytic lymphoma
TEAEs: treatment-related adverse events
AEs: adverse events
ORR: objective response rate
CI: confidence interval
SD: stable disease
PFS: progression-free survival
CR: complete response
PR: partial response
SphK1: sphingosine kinase 1
RES: Resveratrol
EMT: epithelial-mesenchymal transition
RES-Lips: RES-loaded liposomes
HRE: Hemsleya amabilis Diels
